# Open Syntaxin Docks Synaptic Vesicles

**DOI:** 10.1371/journal.pbio.0050198

**Published:** 2007-07-17

**Authors:** Marc Hammarlund, Mark T Palfreyman, Shigeki Watanabe, Shawn Olsen, Erik M Jorgensen

**Affiliations:** 1 Department of Biology, University of Utah, Salt Lake City, Utah, United States of America; 2 Howard Hughes Medical Institute, University of Utah, Salt Lake City, Utah, United States of America; Salk Institute for Biological Studies, United States of America

## Abstract

Synaptic vesicles dock to the plasma membrane at synapses to facilitate rapid exocytosis. Docking was originally proposed to require the soluble N-ethylmaleimide–sensitive fusion attachment protein receptor (SNARE) proteins; however, perturbation studies suggested that docking was independent of the SNARE proteins. We now find that the SNARE protein syntaxin is required for docking of all vesicles at synapses in the nematode Caenorhabditis elegans. The active zone protein UNC-13, which interacts with syntaxin, is also required for docking in the active zone. The docking defects in *unc-13* mutants can be fully rescued by overexpressing a constitutively open form of syntaxin, but not by wild-type syntaxin. These experiments support a model for docking in which UNC-13 converts syntaxin from the closed to the open state, and open syntaxin acts directly in docking vesicles to the plasma membrane. These data provide a molecular basis for synaptic vesicle docking.

## Author Summary

Like Olympic swimmers crouched on their starting blocks, synaptic vesicles prepare for fusion with the neuronal plasma membrane long before the starting gun fires. This preparation enables vesicles to fuse rapidly, synchronously, and in the correct place when the signal finally arrives. A well-known but poorly understood part of vesicle preparation is docking, in which vesicles prepare for release by attaching to the plasma membrane at the eventual site of release. Here, we outline a molecular mechanism for docking. Using a combination of genetics and electron microscopy, we find that docking requires two proteins: the cytoplasmic protein UNC-13 and the plasma membrane protein syntaxin. Syntaxin is known to form two configurations, closed and open. We find that the open form of syntaxin can bypass the docking function of UNC-13, while the closed form cannot. These experiments suggest that docking is the attachment of synaptic vesicles to syntaxin; that syntaxin must be open for this attachment to occur; and that UNC-13′s role in docking is to promote open syntaxin.

## Abstract 3

Experiments in *C. elegans* support a model for synaptic vesicle docking in which the active zone protein UNC-13 converts syntaxin from the closed to the open state, and open syntaxin acts directly in docking vesicles to the plasma membrane.

## Introduction

Fusion of synaptic vesicles with the plasma membrane is thought to occur in three ordered steps: docking, priming, and fusion [1]. The biological state of a synaptic vesicle can be defined by three distinct parameters: morphology (its location in the synapse); physiology (its release competence); and molecular interactions. A goal of studies in neurotransmission is to define the state of the vesicle at each step in exocytosis using morphological, physiological, and molecular criteria. For example, the final step of vesicle fusion, in which vesicles fuse with the plasma membrane, is well defined by these three criteria. Fusing vesicles can be observed by electron microscopy [2,3] and by electrophysiological recordings [4]. The molecular basis of fusion is thought to be mediated by the soluble N-ethylmaleimide–sensitive fusion attachment protein receptors SNARE proteins. When reconstituted into liposomes under permissive conditions, the SNARE proteins have been demonstrated to be necessary and sufficient for membrane fusion [5–12]. Specific sets of complementary SNARE proteins are localized to each cargo vesicle and target compartment in the cell and thereby provide dedicated fusion proteins for each trafficking event [13]. For synaptic vesicle fusion, the vesicular SNARE protein synaptobrevin (also called vesicle-associated membrane protein or VAMP) interacts with the plasma membrane SNARE proteins syntaxin and SNAP-25 to form a four-helix bundle [14]. The formation of this tightly wound structure may provide the driving force for fusion [15–18].

Priming describes a molecular state in which a four-helix SNARE complex has formed between SNARE proteins on a synaptic vesicle and those on the plasma membrane [1]. It is believed that the SNARE proteins partially wind into a complex, but membrane fusion is arrested, and the vesicle is held in this state until triggered to fuse by an increase in calcium [19–24]. Thus, the SNARE proteins function both in priming and in fusion. These primed vesicles are likely to correspond to the physiologically defined readily releasable pool [25].

Docking precedes priming and at this point is defined solely by morphological criteria. Synaptic vesicle docking is observed in electron micrographs of the synapse and is defined as the attachment of vesicles to their target membranes [26–28]. However, the precise definition of docking is a muddle since morphologically docked vesicles are thought to include those in both the primed and unprimed pools [28,29]. Moreover, because standard fixation methods often introduce changes in membrane structure, docking is sometimes defined as including all vesicles near the membrane—usually specified as vesicles within about 30 nm of the membrane [30,31]. Thus, even the morphological definition of docked vesicles varies in the literature.

In addition, the molecular basis for docking is unknown. It is recognized that protein interactions must specifically associate a vesicle to the correct target membrane. In the original SNARE hypothesis, contacts between the SNARE proteins were proposed to confer specificity during docking [32]. However, genetic and other perturbation experiments indicated that SNARE proteins were not required for docking. Disruption of syntaxin, either by mutation [30,33] or by proteolytic cleavage [31,34], dramatically reduced synaptic vesicle fusion, but did not eliminate morphologically docked vesicles. Similarly, in a recent study proteolytic cleavage of syntaxin was found to result in no decrease in docked synaptic vesicles in neurons (although docking of secretory vesicles in neurosecretory cells was reduced) [35]. Thus, the current model for syntaxin function in neurons is that it acts during priming and fusion, after docking has been completed. Although many proteins have defined roles in synaptic transmission, few have been shown to play a role in docking, and none are essential for docking [36].

Here we study docking in the nematode C. elegans using a new fixation method that reduces artifacts [37–39]. We demonstrate that syntaxin is essential for all synaptic vesicle docking, that the syntaxin-binding protein UNC-13 is required for docking vesicles at the active zone, and finally that the docking defects observed in *unc-13* mutants can be bypassed by expressing an open form of syntaxin. Together these data suggest that the open form of syntaxin mediates docking. Thus, all three steps of vesicle fusion—docking, priming, and fusion—depend on the SNARE protein syntaxin.

## Results

### Docking Occurs in Two Distinct Zones

**Figure 1:**
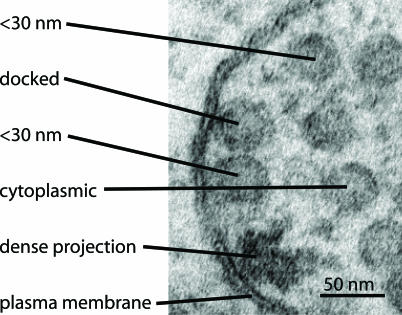
Morphology of Docked Vesicles in C. elegans A neuromuscular junction from a VB acetylcholine motor neuron in a wild-type adult is shown. The center of the synapse is marked by the dense projection. Three distinct morphological classes of vesicles are visible: docked, within 30 nm, and cytoplasmic. Image was acquired at 150 K magnification.

**Figure 2:**
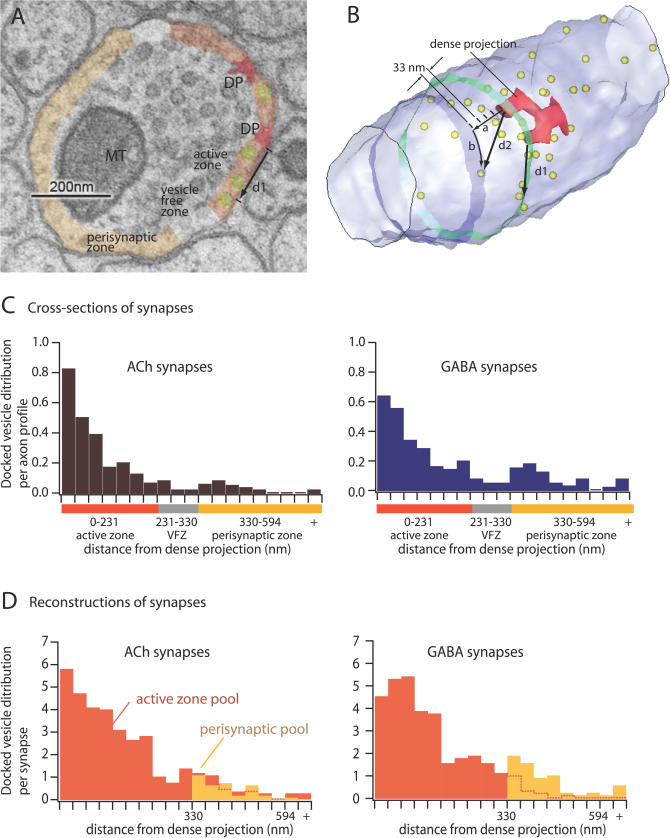
Docking in C. elegans Occurs in Two Zones (A) Shown is a sample electron micrograph from a wild-type animal showing a single VA motor neuron profile in the ventral nerve cord. This profile contains a dense projection (DP), shown in red, which is divided in two in this profile. The membrane region within 231 nm of the dense projection (active zone) is shown in orange, the region from 232–330 nm (VFZ, vesicle-free zone) is light gray, and the region farther than 330 nm from the dense projection (perisynaptic zone) is amber. Yellow, docked synaptic vesicles. MT, mitochondria. The linear distance from the edge of each docked vesicle to the edge of the dense projection was measured (d1). (B) Shown is a reconstruction of a sample VA motor neuron synapse from 38 serial electron micrographs. The plasma membrane (light blue), dense projection (red), and docked vesicles (yellow) are shown. Two single sections are represented by blue and green bands. The green section contains the dense projection and is the image shown in (A). The blue section illustrates the calculation of the distance d2 for docked vesicles in sections without a dense projection. This distance was calculated from the number of 33-nm sections between the vesicle and the dense projection (a) and the radial distance from an imaginary extension of the dense projection to the vesicle (b). (C) Distribution of all docked vesicles in sections containing a dense projection for acetylcholine and GABA neurons. Distances were sorted into 33-nm bins, and the number of measurements in each bin divided by the total number of sections. Data in this analysis are from four wild-type animals comprising 30 synapses and 140 profiles (acetylcholine) and 21 synapses and 100 profiles (GABA). As in (A) the active zone is shown in orange, the region from 232–330 nm (vesicle-free zone) is light gray, and the region farther than 330 nm from the dense projection (perisynaptic zone) is amber. (D) Shown is the distribution of all vesicles in reconstructed synapses for acetylcholine and GABA neurons. Distances were calculated as described above and sorted into 33-nm bins with respect to the distance from the dense projection. For those vesicles in sections not containing dense projections sorting was done based on a tube model of the synapse. Specifically, vesicles were considered in the active zone pool if they were docked within an imaginary stripe extending the length of the tube of a width of 231 nm on either side of the dense projection; these vesicles are indicated in orange. All vesicles not in this stripe, that is those on the far side of the tube, were considered in the perisynaptic zone; these vesicles are indicated in amber. Data in this analysis are from two wild-type animals comprising 11 synapses and 136 profiles (acetylcholine) and nine synapses and 168 profiles (GABA).

To study the ultrastructure of the synapses, we fixed worms using high-pressure freezing followed by substitution of ice by solvent-borne fixatives [38]. We analyzed sections from the ventral nerve cord containing neuromuscular junctions to determine the distribution of synaptic vesicles. In all cases in this study, the wild types were fixed on the same day as the mutant strains and analyzed in parallel, and all genotypes were scored blind. All numerical values and statistical tests are provided in Table S1. In the worm, the acetylcholine neurons in the ventral cord stimulate muscle contraction, and the gamma-aminobutyric acid (GABA) neurons inhibit muscle contraction [40]. The target muscles receive input from numerous en passant synapses, which appear as varicosities containing large numbers of synaptic vesicles abutting the muscle. At each synapse, synaptic vesicles dock to the plasma membrane at sites of release called active zones [41]. Docked vesicles can be identified by visual inspection as vesicles forming a contact patch with the plasma membrane [28,42]. This patch distinguishes them from other vesicles within 30 nm of the membrane that are sometimes identified as “docked” (Figure 1). The active zone flanks an electron-dense specialization called the dense projection (Figures 1 and 2A) [43,44]. We determined the distribution of all docked vesicles relative to the nearest dense projection. In most cases, we defined a synapse as a set of contiguous profiles that contained a dense projection. In these profiles, we measured the distance from the edge of the dense projection to the docked vesicle (Figure 2A and 2B, d1). For the complete reconstruction of the wild-type animal, we also analyzed the adjacent profiles that did not contain a dense projection. In these profiles we calculated the distance between the docked vesicle and the dense projection based on section thickness (Figure 2B, d2).

**Figure 8:**
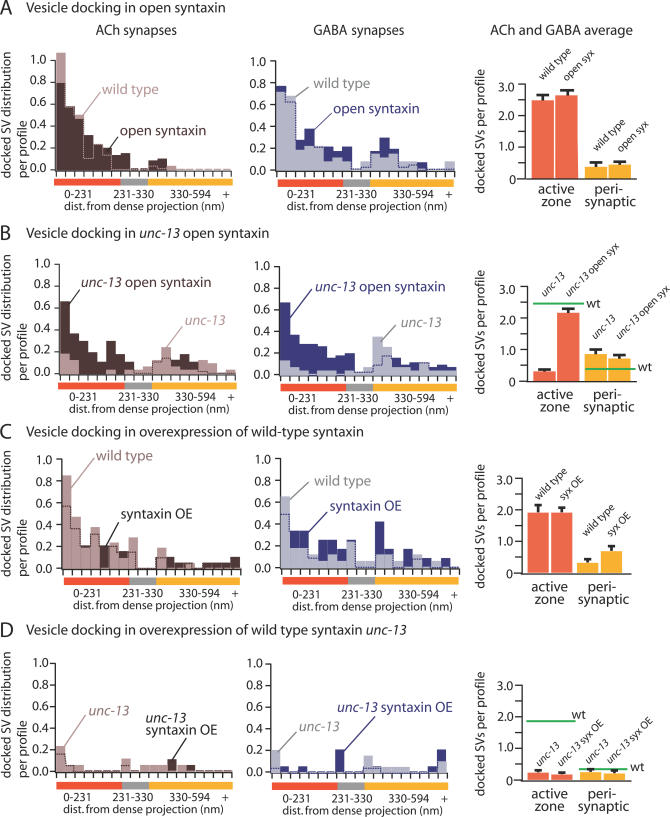
Open Syntaxin Rescues the Docking Defects of *unc-13* Mutants Each histogram shows two distributions: with the wild-type syntaxin allele (light bars) or with syntaxin overexpression (open or wild type, dark bars). Each bar graph shows a comparison of the number of docked vesicles between genotypes in the active zone pool (< 232 nm, orange bars) and the perisynaptic pool (> 300 nm, amber bars). To generate these pools, the number of docked vesicles in each pool was divided by the number of profiles to give a mean value for the number of vesicles in each pool per profile; bars show mean and standard error of mean. The green lines in the bar graphs in (B) and (D) show the number of vesicles in each pool in a matched wild-type control. All experiments that overexpress wild type or open syntaxin are in a syntaxin null genetic background (see Materials and Methods for complete genotypes). Only vesicles in profiles containing a dense projection were included in these analyses. See Table S1 for numbers and statistical analysis. (A) Expression of open instead of wild-type syntaxin does not affect the distribution of docked vesicles. Distribution of docked vesicles in the wild type (N2) and in open syntaxin overexpression in acetylcholine (left) or GABA synapses (right) is shown. For wild-type acetylcholine *n* = 1 animal, seven synapses, and 35 profiles; for *open-syntaxin* acetylcholine *n* = 2 animals, ten synapses, and 57 profiles; for *open-syntaxin* GABA *n* = 1 animal, four synapses, and 28 profiles; and for wild-type GABA *n* = 2 animals, seven synapses, and 49 profiles. (B) Open syntaxin rescues the docking defect in the active zone in *unc-13(s69)*. Green lines indicate docked vesicles in the matched wild-type fixation. For *unc-13* acetylcholine *n* = 2 animals, eight synapses, and 34 profiles; for *unc-13* open syntaxin acetylcholine *n* = 2 animals, ten synapses, and 55 profiles; for *unc-13* GABA *n* = 2 animals, seven synapses, and 33 profiles; for *unc-13 open-syntaxin* GABA, *n* = 2 animals, eight synapses, and 47 profiles. (C) Overexpression of wild-type syntaxin does not affect the distribution of docked vesicles. For wild-type acetylcholine *n* = 1 animal, five synapses, and 21 profiles; for *syntaxin-OE* acetylcholine *n* = 1 animal, five synapses, and 19 profiles; for wild-type GABA *n* = 1 animal, four synapses, and 17 profiles; for *syntaxin-OE* GABA *n* = 1 animal, four synapses, and 12 profiles. (D) Overexpression of wild-type syntaxin does not rescue the docking defect in the active zone pool in *unc-13(s69)*. Green lines indicate docked vesicles in the matched wild-type fixation. For *unc-13(s69)* acetylcholine *n* = 1 animal, five synapses, and 17 profiles; for *unc-13(s69) syntaxin-OE* acetylcholine *n* = 1 animal, five synapses, and 19 profiles; for *unc-13(s69)* GABA *n* = 1 animal, five synapses, and 19 profiles; for *unc-13(s69) syntaxin-OE* GABA *n* = 1 animal, four synapses, and 19 profiles.

Most docked vesicles cluster tightly around the dense projection in the active zone pool. In fully reconstructed synapses there are on average 34.5 docked vesicles in the active zone pool of acetylcholine synapses and 32.6 docked vesicles in the active zone pool of GABA synapses (Figure 2D). Vesicle docking is suppressed in regions lateral to the active zone (Figure 2C and 2D; between 231 and 330 nm from the dense projection). This vesicle-free zone exhibits very little docking in all genotypes analyzed and can be quite pronounced in some datasets (for example, Figure 8). Similar docking-depleted regions have been identified in other synapses [45]. This domain probably corresponds to regions of adhesion [45–48] or endocytosis [3,49–54]. Outside of the vesicle-free zone, on the far side of the synapse, there is a second smaller pool of docked vesicles (Figure 2C and 2D). Such docking is sometimes referred to as ectopic [55]; however since ectopic refers to an abnormal condition, we call this perisynaptic docking. The average number of vesicles in the perisynaptic pool in reconstructed synapses is 3.5 vesicles at acetylcholine synapses and 6.6 vesicles at GABA synapses (Figure 2D). Vesicles in this perisynaptic pool are not oriented toward clear synaptic targets. Although we do not know if such vesicles contain or release neurotransmitter in C. elegans, in vertebrates ectopic release plays an important role in activation of extrasynaptic receptors [55–57]. In summary, vesicles dock to the plasma membrane in at least two domains separated by a docking-suppressed zone.

### Syntaxin Is Not Required for Neuronal Development

Syntaxin null mutants arrest after hatching in the first larval stage [58,59]. To study the loss of syntaxin in adult neurons we generated mosaic strains in the syntaxin null background *unc-64(js115)* (Figure S1). These strains express wild-type syntaxin in the acetylcholine neurons of the head; this expression is required to rescue syntaxin null mutants to adulthood. In *C. elegans,* the ventral body muscles are innervated by the VA and VB acetylcholine motor neurons and the VD GABA motor neurons [60]. We made two mosaic strains: the first lacked expression of syntaxin in both the acetylcholine and GABA motor neurons, (EG3278); the second lacked syntaxin in the GABA motor neurons but expressed syntaxin in the acetylcholine motor neurons (EG3817). The mosaic animals are viable but paralyzed. We confirmed that syntaxin was absent from the relevant motor neurons by immunostaining (Figure S2B and S2C). Importantly, the syntaxin mosaic strains enable us to analyze neurons that lack syntaxin in viable adult animals.

**Figure 3:**
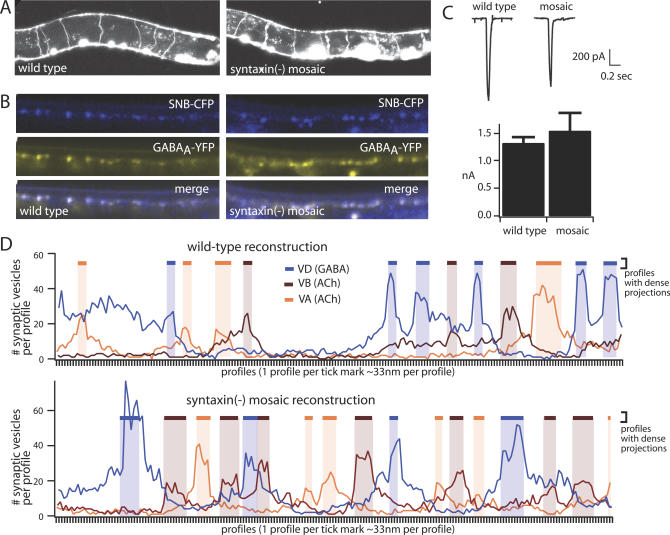
Neurons That Lack Syntaxin Have Normal Morphology (A) GABA neuron development is presented. Sample images of wild type and syntaxin mosaic (EG3278) animals expressing GFP in the GABA neurons are shown. Right, anterior; top, dorsal. In both genotypes, commissures extend at regular intervals from the ventral to the dorsal nerve cord. The bright spots along the ventral nerve cord in the wild type are cell bodies. Cell bodies are also visible in the syntaxin mosaic, as are the larger coelomocytes, which express GFP as a marker for the syntaxin mosaic array. The number of commissures between the dorsal and ventral nerve cords is normal in syntaxin mosaic animals. *n* = 10 adults for each genotype. (B) Pre- and postsynaptic development is presented. Sample images are shown of the dorsal nerve cord of wild-type and syntaxin mosaic (EG3278) animals coexpressing a presynaptic marker (SNB-CFP, synaptobrevin-CFP,) and a postsynaptic marker (GABA_A_ receptor-YFP). SNB-CFP is expressed in GABA neurons, which lack syntaxin in the mosaic animals. Normal colocalization was observed in both genotypes (*n* = 10 adults for each genotype). (C) The postsynaptic receptor field is presented. The postsynaptic response to exogenous GABA is normal in the syntaxin mosaic animals (EG3817) that lack syntaxin in the GABA neurons. Sample traces are shown on the left, and mean and standard error of mean data are shown on the right (*n* = 4 for each for each genotype). (D) EM reconstruction of the nerve cord in syntaxin mosaic animals (EG3817) lacking syntaxin in the GABA motor neurons is presented. The number and distribution of presynaptic specializations and of synaptic vesicle number is normal in syntaxin(−) neurons. The line graphs show the number of vesicles in each serial profile for the wild type (top) and the syntaxin mosaic (EG3817, bottom). Three profiles are presented on each graph: VD, blue; VA, brown; and VB, orange. Profiles containing a dense projection are indicated by a shaded bar of the corresponding color. The distribution of the dense projections in GABA syntaxin(−) neurons is similar to the wild-type pattern, with inhibitory and excitatory synapses alternating along the length of the nerve cord. Reconstructions are from 201 serial sections for the wild type and 199 serial sections for the mosaic strain.

Loss of syntaxin function could result in abnormal development or cell death. To determine whether development was normal, we assayed the structure of the syntaxin mutant neurons by expressing green fluorescent protein (GFP) in the GABA neurons (Figure 3A). The number of GABA neurons and arrangement of commissures is normal in the mosaic animals (syntaxin mosaic: 16.8 GABA commissures/animal; wild type: 16.8 GABA commissures/animal; no abnormalities were observed; the large cells in the mosaic are coelomocytes that express GFP to mark the transgene). We also assayed the density of synaptic varicosities of syntaxin mutant neurons by tagging synaptic vesicles in the GABA neurons with synaptobrevin-GFP (Figure 3B). The number of synapses in these cells is similar to the wild type (syntaxin mosaic: 1.9 varicosities/10 μm; wild type: 2.3 varicosities/10 μm) (see Materials and Methods). Postsynaptic GABA receptors cluster normally on the muscle opposite GABA presynaptic varicosities in the syntaxin mosaic (Figure 3B). The clustered postsynaptic GABA receptors are functionally indistinguishable from those in wild-type animals (response to GABA application in syntaxin mosaic: 1.53 ± 0.33 nA; wild type: 1.31 ± 0.11 nA; *p* = 0.54) (Figure 3C). Finally, we confirmed that these synaptic contacts are intact at the ultrastructural level, and that the interweaving of acetylcholine and GABA neuromuscular junctions is normal (Figure 3D). These results differ from *Drosophila* in which syntaxin mutants exhibit developmental abnormalities [30,61–63]. In the fly there is a substantial maternal contribution of syntaxin to the embryo that provides important functions during cellularization [61,63]. In mutants lacking zygotic expression of syntaxin, fewer boutons are observed, and in late embryos the postsynaptic clusters of neurotransmitter receptors apparently dissipate [30,63,64–66]. In the fly studies, the entire embryo lacked syntaxin; thus, some of these defects may not be cell autonomous. In the mosaic worm, the absence of syntaxin in the GABA neurons does not lead to degeneration of presynaptic or postsynaptic elements.

### Syntaxin Is Required for Synaptic Vesicle Exocytosis

**Figure 4:**
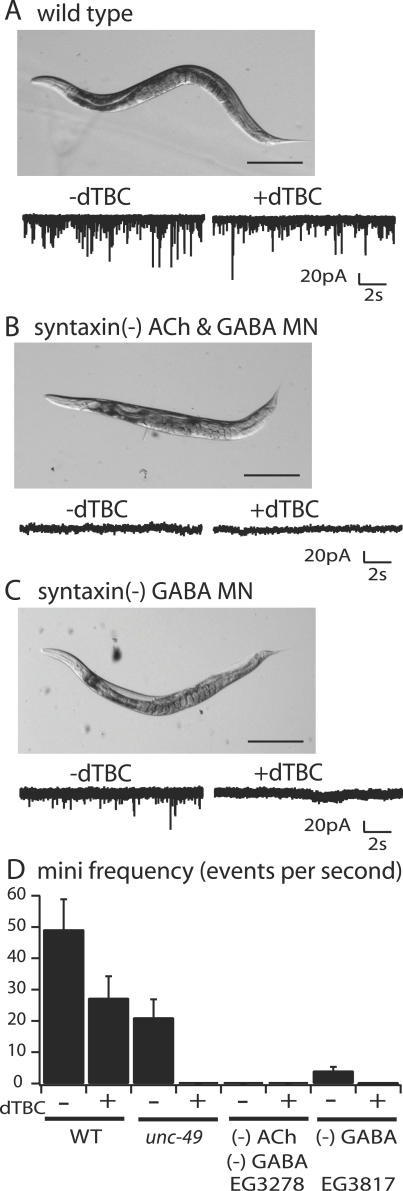
Syntaxin Is Required for Synaptic Vesicle Exocytosis Shown is endogenous activity in (A) the wild type, (B) syntaxin mosaic EG3278, and (C) syntaxin mosaic EG3817 before and after the addition of d-tubocurare (dTBC). EG3278 lacks syntaxin in both the acetylcholine and GABA neurons, while EG3817 lacks syntaxin in the GABA neurons. Before the addition of d-tubocurare, activity represents input from acetylcholine and GABA neurons. (D) Before the addition of d-tubocurare, wild-type animals exhibited 42.8 ± 6.5 fusions per second, EG3278 animals 0.02 ± 0.01 fusions per second (*p* < 0.0001), and EG3817 animals 4.3 ± 1.1 (*p* = 0.0016). After the addition of d-tubocurare, activity represents input from GABA neurons only. Under these conditions, wild-type animals exhibited 28.5 ± 4.9 fusions per second, EG3278 animals 0.00 ± 0.00 fusions per second (*p* < 0.0001), and EG3817 0.06 ± 0.03 (*p* < 0.0001). d-tubocurare blocks all acetylcholine minis, since the drug blocks all minis in *unc-49(e407)* mutants, which lack the GABA_A_ receptor [69] (21.0 ± 5.8 fusions per second before treatment and 0.0 ± 0.0 fusions per second after treatment). Recordings were performed in 5 mM Ca^2+^; *n* = 8 for the wild type, *n* = 2 for EG3278, and *n* = 5 for EG3817. Scale bars in photographs, 200 μm.

Previous experiments demonstrated that syntaxin is required for synaptic vesicle exocytosis [30,31,34,62]. Similarly, we observe that syntaxin is required for exocytosis in the nematode. In C. elegans, individual synaptic vesicle fusions can be observed by recording miniature postsynaptic currents (minis) in the postsynaptic muscles [67]. Under our recording conditions acetylcholine and GABA miniature currents are both inward and are of roughly the same amplitude (combined rate: 42.8 ± 6.5 fusions per second) (Figure 4A) [67]. By adding d-tubocurare we can block acetylcholine receptors and monitor synaptic vesicle exocytosis from only the GABA motor neurons (GABA rate: 28.5 ± 4.8 fusions per second) (Figure 4A and 4D). d-tubocurare is completely effective at blocking all acetylcholine-induced currents, since it eliminates all minis in mutants lacking the muscle GABA receptor UNC-49, *unc-49(e407)* (21.0 ± 5.8 fusions per second before treatment; 0.0 ± 0.0 fusions per second after treatment) (see d-tubocurare in Materials and Methods) (Figure 4D). To determine if syntaxin is required for synaptic vesicle exocytosis, we recorded from syntaxin mosaic animals. The EG3278 mosaic animals almost completely lack mini currents from both the acetylcholine and GABA neurons (Figure 4B and 4D) (Acetylcholine 0.02 ± 0.01 fusions per second; GABA 0.00 ± 0.00 fusions per second). Thus, syntaxin is required for exocytosis at both excitatory acetylcholine synapses and inhibitory GABA synapses.

The requirement for syntaxin in exocytosis could be cell intrinsic. Alternatively, *unc-49(e407)* syntaxin(−) motor neurons might fail to release synaptic vesicles because they are not excited by upstream neurons. To control for this possibility, we assayed transmission in the second syntaxin mosaic strain (EG3817) that expresses syntaxin in the acetylcholine motor neurons but lacks syntaxin in the GABA motor neurons (Figure S1). These animals are viable and healthy but exhibit behavioral defects associated with loss of GABA neurotransmission. Specifically, EG3817 animals shrink when touched due to lack of GABA inhibition of the body muscles [68,69] and are constipated due to loss of activation of a GABA-gated cation channel during defecation [70]. The syntaxin-expressing acetylcholine neurons exhibit substantial levels of vesicle fusion (Figure 4C and 4D) (4.3 ± 1.1 fusions per second). Thus, the lack of exocytosis in syntaxin(−) cells is due to a cell-autonomous requirement for syntaxin rather than due to the paralysis of the mutant strain. By contrast, the mini rate in the syntaxin(−) GABA neurons is 1% of the rate in the syntaxin(+) acetylcholine neurons (Fig 4C and 4D; 0.06 ± 0.03 fusions per second). GABA neurons receive inputs from the acetylcholine motor neurons. Restoring acetylcholine inputs into the GABA motor neurons did not rescue exocytosis; thus, the observed defects are not due to a lack of synaptic input into the motor neurons. Note that synaptic activity is not fully rescued in the acetylcholine neurons; mini frequency is only 20% compared to the wild type. There are two possible causes for the lack of complete rescue: either syntaxin is not expressed at high levels in these cells, or modulatory inputs from other neurons, which are missing in the mosaic, are required to obtain normal levels of activity from these synapses.

### Syntaxin Is Essential for All Synaptic Vesicle Docking

**Figure 5:**
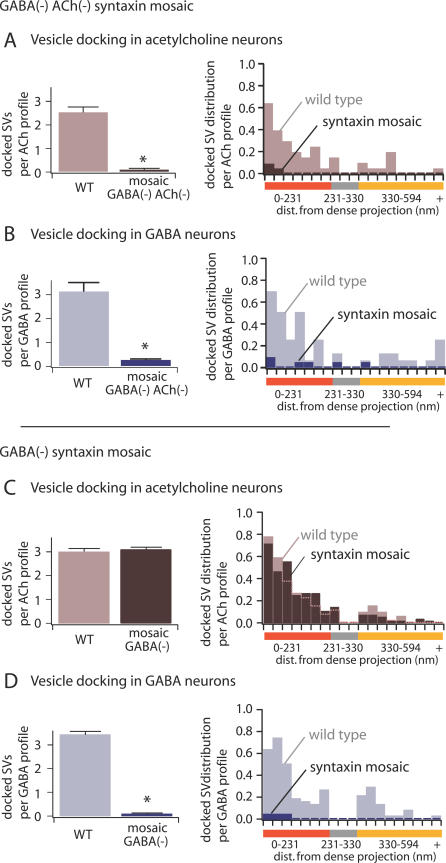
Syntaxin Is Essential for Synaptic Vesicle Docking Each row shows two comparisons: total docked vesicles and the distribution of docked vesicles. For total docked vesicles (left), the mean number of docked vesicles per profile was calculated for each synapse (see Table S1 for complete methods and results). Bars show mean and standard error of the mean; *, *p*-values < 0.001. For vesicle distributions (right), the distance from the dense projection to each docked vesicle was determined, and these measurements were sorted into 33-nm bins. The number of vesicles in each bin was divided by the number of profiles to yield an average number of vesicles per profile in each bin. For both comparisons, only vesicles in profiles containing a dense projection were included. (A) Shown is a comparison of acetylcholine neurons in wild-type animals and mosaic animals with reduced syntaxin in the GABA and acetylcholine neurons (EG3278). Wild type *n* = 1 animal, five synapses, and 20 profiles and mosaic *n* = 1 animal, five synapses, and 24 profiles. (B) Shown is a comparison of GABA neurons in wild-type animals and mosaic animals with reduced syntaxin in the GABA and acetylcholine neurons (EG3278). Wild type *n* = 1 animal, four synapses, and 16 profiles and mosaic *n* = 1 animal, four synapses, and 22 profiles. (C) Shown is a comparison of acetylcholine neurons in wild-type animals and mosaic animals lacking syntaxin in the GABA neurons (EG3817). Wild type *n* = 2 animals, ten synapses, and 53 profiles and mosaic *n* = 2 animals, 11 synapses, and 66 profiles. (D) Shown is a comparison of GABA neurons in wild-type animals and mosaic animals (EG3817). Wild type *n* = 2 animals, eight synapses, and 38 profiles and mosaic *n* = 2 animals, ten synapses, and 51 profiles.

Syntaxin is not thought to function in synaptic vesicle docking [30,31,34,35]; however, syntaxin is known to mediate interactions between the plasma membrane and synaptic vesicles that could in principle dock vesicles. To determine whether loss of syntaxin affects synaptic vesicle docking, we fixed the syntaxin mosaic strains by high-pressure freezing and analyzed them by serial section electron microscopy. An analysis of the distribution of vesicles at synaptic profiles in the mosaic animals demonstrated that syntaxin is required for synaptic vesicle docking. First, we analyzed docking in the EG3278 syntaxin mosaic, which lacks syntaxin in both acetylcholine and GABA neurons. These mosaic animals exhibit a severe reduction of docking in the acetylcholine neurons (docked vesicles per acetylcholine synaptic profile: mosaic 0.12 ± 0.05; wild type 2.56 ± 0.22; *p* < 0.0001; see Table S1 for statistical methods and complete list of *p*-values) (Figure 5A) and in the GABA neurons (docked vesicles per profile: mosaic 0.27 ± 0.04; wild type 3.13 ± 0.33; *p* = 0.0001) (Figure 5B). Thus, syntaxin is required for docking at both excitatory acetylcholine synapses and inhibitory GABA synapses. Second, to confirm that the docking defect in syntaxin(−) neurons is cell autonomous, we examined docking in the EG3817 syntaxin mosaic. In this strain, docking at acetylcholine synapses in mosaic animals is fully rescued compared to wild-type synapses (docked vesicles per acetylcholine synaptic profile: syntaxin mosaic 3.09 ± 0.11; wild type 2.99 ± 0.15; *p* = 0.59) (Figure 5C). By contrast, in the syntaxin(−) GABA neurons of the same strain, docked vesicles are reduced to 3% compared to wild-type synapses (docked vesicles per GABA synaptic profile: syntaxin mosaic 0.09 ± 0.05; wild type 3.42 ± 0.15; *p* < 0.0001) (Figure 5D). The full rescue of docking in acetylcholine synapses of the mosaic strain confirms that the docking defects are cell autonomous and do not result from general paralysis. In all syntaxin (−) neurons analyzed, docking was eliminated both in the active zone pool as well as the perisynaptic pool; thus, both of the docked pools require syntaxin.

**Figure 6:**
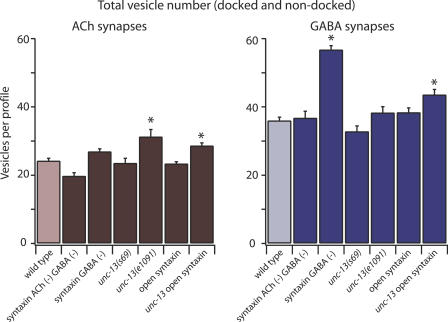
Total Synaptic Vesicles Are Not Reduced in Syntaxin or *unc-13* Mutants The average number of synaptic vesicles in single profiles containing a dense projection for each genotype is shown. Both undocked and docked vesicles were included in this analysis. Left, total vesicles for acetylcholine synaptic profiles; right, total vesicles for GABA synaptic profiles (Table S1 for complete list of *p*-values). Bars show mean and standard error of the mean; *, *p*-values < 0.001 compared to wild type. Note that there is an unusually large increase in vesicle number at the GABA synapses of one of the mosaic syntaxin strains (EG3817). This increase is not observed at synapses of other syntaxin(−) genotypes.

This defect in docking was not caused by a lack of vesicles at the synapse. In both mosaic strains, the total vesicle number was not reduced (Figure 6). In addition, the distribution of this reserve pool of vesicles was normal (Figure S3); vesicles were clustered near the dense projection in the synaptic varicosity. These data suggest that the docking defect in syntaxin mutant neurons is not the result of a general trafficking defect such as synaptic vesicle biogenesis, transport, or clustering.

### UNC-13 Is Required for Vesicle Docking in the Active Zone

**Figure 7:**
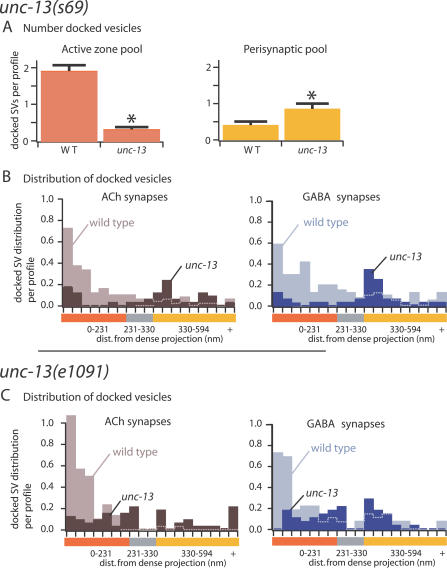
UNC-13 Is Required for Docking in the Active Zone (A and B) Docked vesicles in the wild type and *unc-13(s69)* are presented. Only vesicles in axon profiles containing a dense projection were included in these analyses. (A) Docked vesicles from acetylcholine and GABA synapses combined are shown. Docking in the active zone pool (orange) is greatly reduced while docking in the perisynaptic pool (amber) is increased in *unc-13(s69)* mutants. (B) Distribution of docked vesicles in the wild type and *unc-13(s69)* in acetylcholine (left) or GABA synapses (right) is shown. For wild-type acetylcholine *n* = 2 animals, 13 synapses, and 52 profiles; for *unc-13* acetylcholine *n* = 2 animals, eight synapses, and 34 profiles; for wild-type GABA *n* = 2 animals, nine synapses, and 42 profiles; and for *unc-13* GABA *n* = 2 animals, seven synapses, and 33 profiles. (C) Distribution of docked vesicles in the wild type and *unc-13(e1091)* in acetylcholine (left) or GABA synapses (right) is shown. For wild-type acetylcholine *n* = 1 animal, seven synapses, and 35 profiles; for *unc-13* acetylcholine *n* = 1 animal, seven synapses, and 33 profiles; for wild-type GABA *n* = 1 animal, four synapses, and 20 profiles; and for *unc-13* GABA *n* = 1 animal, four synapses, and 28 profiles.

UNC-13 is a syntaxin-binding protein that is required for synaptic vesicle priming [71–73]. To determine whether UNC-13 functions in docking at specific membrane domains, we analyzed the number of docked vesicles in the active zone and perisynaptic pools in *unc-13* mutants. The number of docked vesicles in the active zone pool in *unc-13* mutants is 16% that of the wild type (docked vesicles in the active zone per profile: *unc-13* = 0.31 ± 0.06; wild type = 1.91 ± 0.16; *p* < 0.0001) (Figure 7A and 7B). Docking in the perisynaptic pool actually increases slightly in *unc-13* (docked vesicles in the perisynaptic zone per profile: *unc-13* = 0.85 ± 0.15; wild type = 0.41 ± 0.10; *p* = 0.01). These results differ from our previous results using ice-cold glutaraldehyde fixations [73]. In those experiments we combined active zone regions with peri-synaptic regions, which could obscure decreases in active zone docking. Moreover, we defined the docked pool as vesicles within 30 nm of the membrane. When we apply those criteria to the current dataset, we also do not observe a decrease in docking (see Materials and Methods). In addition, our current results are in agreement with data from two independent laboratories [54,74].

To demonstrate that the docking defects were not caused by irrelevant background mutations we analyzed a second allele, *unc-13(e1091)*. Similar results were obtained with this mutant: decreased docking was observed in the active zone pool and increased docking in the perisynaptic pool (active zone 28%, perisynaptic zone 145% compared to the wild type) (Figure 7C). The decrease in docking is restricted to the active zone and is most severe near the dense projection. The specific reduction in docking in the active zone pool is consistent with the localization of UNC-13 near the dense projection [54].

Surprisingly, we did not observe an increase in the number of cytoplasmic vesicles in *unc-13* mutant animals (Figure 6), despite observing an increase using a different fixation protocol [73]. In the present study *unc-13* and other release-defective genotypes generally do not display an increase in cytoplasmic vesicle number (Figure 6). This lack of increase in the number of cytoplasmic vesicles in *unc-13* mutant animals was also found in an independent study using high pressure freezing [74]. Glutaraldehyde fixations used in previous studies can induce vesicle fusion [75]. Glutaraldehyde-induced fusion would result in a reduction of docked vesicles in the wild type relative to release-defective mutants and thus lead one to believe that there is an actual accumulation in the mutant. We have confirmed these differences by comparing glutaraldehyde and freeze-substituted fixations in parallel (see Materials and Methods). It is still possible that synaptic vesicles accumulate in the reserve pool of *unc-13* mutants. These data only analyze synaptic vesicles in profiles containing a dense projection; the reserve pool was not fully reconstructed.

### Open Syntaxin Bypasses the Requirement for UNC-13 in Docking

Syntaxin can adopt two configurations: a closed configuration in which the N-terminal Habc domain binds to the SNARE motif and an open configuration in which this *cis* interaction does not occur. Mutations in the linker between the Habc domain and the SNARE motif cause syntaxin to preferentially adopt the open conformation [76]. We found that the replacement of wild-type syntaxin with the open form of syntaxin does not lead to a redistribution of docked vesicles (docked vesicles per profile: open syntaxin 3.27 ± 0.21; wild type 2.92 ± 0.21; *p* = 0.27) (Figure 8A).

It has previously been proposed that UNC-13 opens or maintains the open state of syntaxin at the active zone [77]. Since docking requires syntaxin, this suggests that the docking defects in *unc-13* animals might be due to its failure to open syntaxin. To test this idea, we examined docking in *unc-13* mutant animals in which wild-type syntaxin was replaced with open syntaxin. We found that expression of the open form of syntaxin fully rescues the docking defect in *unc-13(s69)* mutants (docked vesicles per active zone profile: *unc-13 open-syntaxin* 2.18 ± 0.14; wild type 2.48 ± 0.17; *p* = 0.19) (Figure 8B). To control for the possibility that this result was due to overexpression of the syntaxin protein rather than its conformation, we tested whether overexpression of wild-type syntaxin could restore docking to *unc-13* mutants. First, overexpression of wild-type syntaxin had no effect on the distribution of docked vesicles in an otherwise wild-type background (Figure 8C). Second, overexpression of wild-type syntaxin had no effect on docking in *unc-13* animals (docked vesicles per active zone profile: *unc-13* syntaxin OE 0.19 ± 0.05; wild type 1.93 ± 0.23; *p* < 0.0001) (Figure 8D). Thus, the function of UNC-13 in vesicle docking is specifically to promote the open state of syntaxin. Finally, the full bypass of *unc-13* mutants by open syntaxin demonstrates that syntaxin functions in docking downstream of UNC-13, further reinforcing the fact that syntaxin plays a direct role in docking rather than an indirect role in trafficking or development.

Interestingly, the distribution of docked vesicles is normal in the presence of open syntaxin (Figure 8A). Thus, it is likely that open syntaxin is involved in the mechanics of docking but not in the distribution of docked vesicles. Similarly, this distribution is independent of UNC-13, since the distribution of docked vesicles is normal in the *unc-13* open syntaxin genotype. Other proteins must therefore determine the distribution of docked vesicles relative to the dense projection. Mutants lacking tomosyn, for example, have a large increase in the number and distribution of docked synaptic vesicles [74].

### Docked Vesicles Are Release Competent in the Absence of UNC-13

As described above, normal vesicle docking occurs in the absence of UNC-13 when open syntaxin is present. We tested the release competence of these vesicles by comparing spontaneous and evoked release in *unc-13* mutant animals in the presence and absence of open syntaxin. Experiments were performed at two different concentrations of external calcium. We found that open syntaxin restores vesicle fusion to approximately one-third wild-type levels.

**Figure 9:**
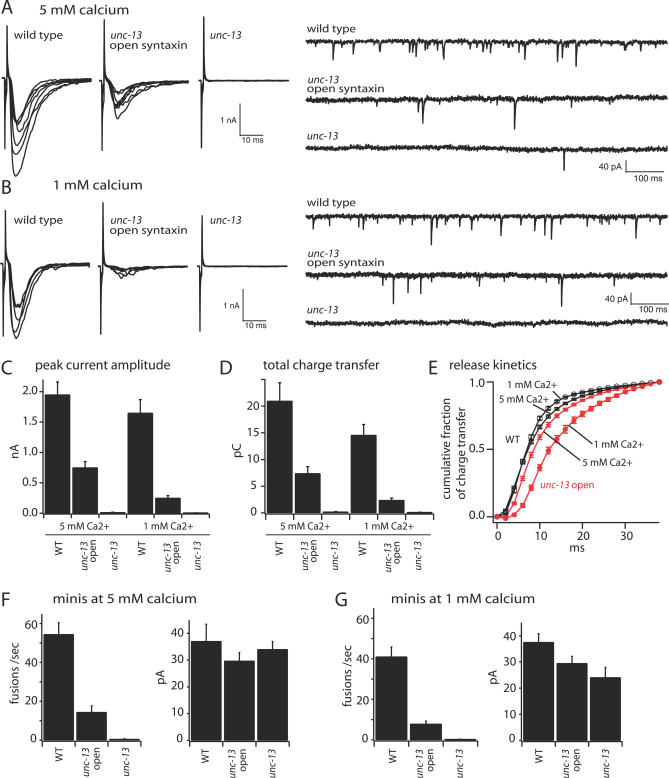
Docked Vesicles Are Release Competent in the Absence of UNC-13 All experiments that overexpress wild type or open syntaxin are in a syntaxin null genetic background (see Materials and Methods). Error bars represent standard error of mean in all cases. (A–D) Open syntaxin bypasses evoked response defects of *unc-13* mutants. All traces for wild type, *unc-13 open-syntaxin*, and *unc-13* evoked responses (left) are shown (A) at 5 mM calcium and (B) and at 1 mM calcium. (C) The mean peak amplitude for evoked responses is: at 5 mM calcium (wild type 2.0 ± 0.2 nA, *n* = 7; *unc-13 open-syntaxin* 0.8 ± 0.1 nA, *n* = 7; *unc-13* 0.015 ± 0.004 nA, *n* = 9) and at 1 mM calcium (wild type 1.7 ± 0.2 nA, *n* = 6; *unc-13 open-syntaxin* 0.25 ± 0.04 nA, *n* = 6; *unc-13* 0.005 ± 0.003 nA, *n* = 8). (D) Mean charge transfer evoked is: at 5 mM calcium (wild type 21.0 ± 3.4 pC, *n* = 7; *unc-13 open-syntaxin* 7.4 ± 1.3 pC, *n* = 7; *unc-13* 0.22 ± 0.04 pC, *n* = 9) and at 1 mM calcium (wild type 14.6 ± 2.0 pC, *n* = 6; *unc-13 open-syntaxin* 2.4 ± 0.4 pC, *n* = 6; *unc-13* 0.10 ± 0.03 pC, *n* = 8). (E) Evoked responses are delayed and asynchronous in *unc-13 open-syntaxin* animals. The cumulative plot for the fraction of total charge transfer as a function of time after the beginning of the stimulus artifact is shown. Individual points represent average cumulative current transfer at 2-ms intervals for each genotype. *unc-13 open-syntaxin* animals (in red) show a delay in release during evoked response at 1 mM calcium. (A and B) (F and G) Open syntaxin restores endogenous release (minis) in *unc-13* mutants. (A) Right, representative traces of endogenous activity are shown in the wild type, *unc-13 open-syntaxin*, and *unc-13* at 5 mM calcium. (F) Left, mean mini frequency at 5 mM calcium is shown (wild type 54.5 ± 6.0 fusions per second, *n* = 6; *unc-13 open-syntaxin* 14.4 ± 3.3 fusions per second, *n* = 6; *unc-13* 0.6 ± 0.1 fusions per second, *n* = 9). Right, mean mini amplitude is not altered in mutants (wild type 37.1 ± 6.3 pA, *n* = 6; *unc-13 open-syntaxin* 29.7 ± 3.1 pA, *n* = 6; *unc-13* 34.0 ± 2.9 pA, *n* = 9). (B) Right, representative traces of endogenous activity in the wild type, *unc-13 open-syntaxin*, and *unc-13* at 1 mM calcium. (G) Left, mean mini frequency at 1 mM calcium (wild type 41.1 ± 4.8 fusions per second, *n* = 12); *unc-13 open-syntaxin* 7.9 ± 1.4 fusions per second, *n* = 13; *unc-13* 0.3 ± 0.1 fusions per second *n* = 8). Right, mean mini amplitude is not altered in mutants (wild type 37.5 ± 3.3 pA, *n* = 6; *unc-13 open-syntaxin* 29.5 ± 2.7 pA, *n* = 6; *unc-13* 24.1 ± 3.8 pA, *n* = 9).

First, we examined vesicle fusion in external solutions containing 5 mM calcium. In *unc-13(s69)* animals evoked responses were essentially absent (0.015 ± 0.004 nA; *n* = 9) (Figure 9A and 9C). However, expression of open syntaxin in *unc-13(s69)* animals partially rescued the evoked response (Figure 9A). Peak amplitude was restored to 38% of wild type (*unc-13 open-syntaxin* 0.75 ± 0.10 nA, *n* = 7; wild type 1.95 ± 0.21, *n* = 7) (Figure 9C), and total current transferred was restored to 35% of wild type (*unc-13 open-syntaxin* 7.41 ± 1.26 pC, *n* = 7; wild type 20.98 ± 3.41 pC, *n* = 7) (Figure 9D). In addition to the rescue of evoked responses, endogenous fusion events were restored to 26% of wild type (*unc-13* 0.59 ± 0.13 Hz, *n* = 9; *unc-13 open-syntaxin* 14.42 ± 3.25 Hz, *n* = 6; wild type 54.47 ± 6 Hz, *n* = 6) (Figure 9A and 9F). Thus, docking via open syntaxin in the absence of UNC-13 results in vesicles that can be released, although release is not restored to wild-type levels.

Second, we examined vesicle fusion in external solutions containing 1 mM calcium. Again, the presence of open syntaxin partially rescued the *unc-13* defects in both evoked and endogenous release (Figure 9B–9D and 9G). However, this experiment revealed additional characteristics of vesicle fusion in *unc-13 open-syntaxin* animals. The evoked response in wild-type animals at 1 mM calcium was only 15% lower than the response at 5 mM calcium (1.95 ± 0.21 nA at 5 mM calcium, *n* = 7; 1.65 ± 0.22 nA at 1 mM calcium, *n* = 6) (Figure 9B and 9C). By contrast, evoked responses in *unc-13* open syntaxin at 1 mM calcium were 67% lower than the response at 5 mM calcium (0.75 ± 0.10 nA at 5mM calcium, n = 7; 0.25 ± 0.04 nA at 1 mM calcium, n = 6) (Figure 9B and 9C). Further, release kinetics were altered in *unc-13 open-syntaxin* animals at 1 mM calcium: release was slower and more asynchronous in comparison to wild-type animals (Figure 9B and 9E).

## Discussion

Previously, syntaxin was not thought to be required for docking. By contrast, our results demonstrate that syntaxin is required for docking synaptic vesicles at the C. elegans neuromuscular junction. Vesicles are docked in two pools: the active zone pool and the perisynaptic pool. We also find that UNC-13 is required for synaptic vesicle docking. However, while both pools of docked vesicles depend absolutely on syntaxin, UNC-13 only plays a role at the active zone. Finally, the docking function of UNC-13 is completely bypassed by open syntaxin.

The observed docking defects in the syntaxin and *unc-13* mutant synapses are likely to be caused by a direct role of these proteins in the docking pathway rather than by an indirect effect on neuronal health. First, syntaxin acts cell autonomously: expressing syntaxin in the acetylcholine neurons rescues docking in these cells but not in downstream neurons in the motor circuit. Second, chronic lack of syntaxin does not lead to developmental abnormalities in the cell. Synaptic vesicles and synaptic vesicle components such as synaptobrevin are transported to the synapse, vesicles are clustered, dense projections and adherens junctions appear normal at the ultrastructural level, the postsynaptic receptors cluster appropriately, and the receptors are functional: the synapses appear to be intact. Third, syntaxin appears to play a late role in docking. The syntaxin-binding protein UNC-13 is required for docking as well, and open syntaxin can rescue the docking defect in *unc-13* mutants, suggesting that syntaxin acts downstream of UNC-13 during docking. These data are most consistent with a direct role for syntaxin in the docking of synaptic vesicles.

A role for syntaxin in docking conflicts with previous studies [30,31,34,35]. It is unlikely that syntaxin function is not conserved among organisms; it is more likely that the conflicting results arise from the difficulties in studying docking. The different conclusions might be attributed to two causes: definitions for docking and the potential for residual syntaxin. First, different definitions for docking were used in these various studies. In the present study only vesicles contacting the membrane were considered docked (Figure 1). This definition was used in studies of vertebrate synapses comparing the docked and readily releasable pools [26,27,42]. By contrast, previous syntaxin studies, as well as our previous UNC-13 studies, defined docked vesicles as those near the plasma membrane (less than 30, 40, or 50 nm, [30,31,73]). If we analyze our current data using the 30 nm definition, we also do not detect decreases in docking (for example, vesicles within 30 nm per profile, matched wild-type GABA 5.6 ± 0.2; syntaxin(−) GABA from EG3817 5.4 ± 0.3; *p* = 0.49). It was not possible to reanalyze our previous data with our current definition of docking, because the glutaraldehyde fixation used in the previous experiments did not preserve membranes well enough to distinguish between docked and undocked vesicles. Tethering proteins span larger distances than the SNARE proteins and thus are thought to function in those vesicles that are close to but not contacting the plasma membrane [78,79]. Our data thus suggest that syntaxin is not required to tether synaptic vesicles to the membrane. In contrast to synaptic vesicles, secretory vesicles require syntaxin for tethering [35,80].

The second possible explanation for the discrepancy is that residual syntaxin could have mediated docking in previous experiments. In the studies on squid and cultured hippocampal cells, syntaxin was acutely disrupted by protease digestion; nevertheless, about 10% of synaptic vesicle fusions remained, suggesting that some syntaxin was still present [31,34,35]. Further, syntaxin may itself be redundant, in agreement with the almost complete lack of a phenotype in syntaxin knockout mice [81]. Studies in *Drosophila* used mutation rather than protease cleavage to disrupt syntaxin. In fly syntaxin mutants, vesicle fusions were 5% the wild-type rate [30]; much greater than the fusion rate observed in the syntaxin mosaics in C. elegans (less than 0.2% of the wild-type rate). In *Drosophila*, there is a significant maternal contribution of syntaxin [61,63], and it has been suggested that syntaxin might perdure until late embryogenesis [30,33]. In our own data, although syntaxin is not detectable by antibody staining, we do observe a few docked vesicles and a few spontaneous fusions (Figures 4 and 5). These rare events are likely due to residual syntaxin, either as a result of read-through of the stop codon in *unc-64(js115)* or as a result of misexpression from our rescuing array. Thus, syntaxin is likely to be essential for all synaptic vesicle docking.

In addition to syntaxin, docking in the active zone also relies on UNC-13. The docking defect in *unc-13* mutants is completely bypassed by open syntaxin but not by closed syntaxin. This observation suggests that UNC-13′s function in docking is to promote open syntaxin. However, open syntaxin does not completely restore exocytosis in *unc-13* mutant animals. Specifically, in *unc-13* mutants expressing open syntaxin evoked response is 38% of the wild type. Further, we find that the presence of open syntaxin only slightly improves locomotion in *unc-13* mutants (unpublished data). The simplest explanation is that UNC-13 has a second function after docking to increase the probability of fusion [82,83]. Alternatively, levels of open syntaxin might not be sufficient to support normal exocytosis in the absence of UNC-13. It is worth noting that this strain has changed with time; previously the strain was more active and evoked responses were more robust [77]. By contrast, some recently derived strains have no evoked response [84]. It is possible that expression levels have declined in these strains. We propose that only a few molecules of open syntaxin suffice for docking a vesicle, but that multiple molecules of open syntaxin are required to mediate normal exocytosis. Thus, very high expression levels of open syntaxin might be required to bypass the function of UNC-13. In a wild-type synapse, UNC-13 is specifically localized to active zones [54], where it can locally generate the high levels of open syntaxin that are required for release.

How does open syntaxin interact with synaptic vesicles during docking? There are two regions of syntaxin that could be involved: the Habc domain and the SNARE motif. In the open state of syntaxin both of these regions are free to interact with vesicle proteins. It is possible that the Habc domain mediates docking independently of SNARE function. In this model, the other SNARE proteins would not be required for docking. In support of this idea, previous data suggest that genetic and toxin disruption of synaptobrevin and SNAP-25 does not disrupt docking [30,80,85–87]. However, these studies used differing definitions of vesicle docking, perhaps obscuring specific docking defects. Further, it has been suggested that redundant SNARE proteins compensate for the loss of the synaptic SNAREs in these experiments [81,85,87–90]. If the SNARE motif of syntaxin mediates docking then it is likely that the SNARE proteins synaptobrevin and SNAP-25, which interact with the SNARE motif of syntaxin, will also be required for docking. In this case, formation of the SNARE complex would mediate docking, as originally predicted in the SNARE hypothesis [32], and the distinction between morphological docking and priming would not exist.

## Materials and Methods

### Terminology.

A synapse is defined as the serial profiles containing a dense projection and usually comprised three to four adjacent profiles. The exception is the complete wild-type reconstruction described in Figure 1, in which a synapse included all the profiles on either side of the dense projection up to the profile on either side where the synaptic vesicle number fell to the average intersynaptic vesicle density, as determined from all the profiles analyzed. The dense projection is defined as an electron dense structure in the center of the active zone [43,44]. In *C. elegans,* this structure is quite prominent compared to many vertebrate central nervous system synapses [91]. The active zone encompasses the region where synaptic vesicles are docked opposite the postsynaptic target [41]. In our micrographs, docked vesicles extended laterally an average of 230 nm from the dense projection. Docked vesicles are morphologically defined as those contacting the plasma membrane [27,42]. In this study, vesicles were considered docked if their membranes and those of the plasma membrane appeared to be in direct contact (see Figure 1). The perisynaptic docked pool includes any docked vesicles not in the active zone. These can be oriented away from the active zone and would presumably not be part of the physiologically defined readily releasable pool.

### Plasmids.

To drive the expression of syntaxin/UNC-64 under exogenous promoters, a minigene cassette (pMH421) was constructed that contains the endogenous *unc-64* promoter, the ATG, an inserted SphI site, *unc-64* cDNA up to the NheI site (in exon 6), followed by genomic sequence including the 3′ UTR (Figure S1). This construct was injected and rescued the *unc-64(js115)* null phenotype (unpublished data). Next, the endogenous *unc-64* promoter was replaced with the *unc-17, rab-3,* and *glr-1* promoters, which were amplified by PCR. For *unc-17,* the primers were *unc-17* 5′ and *unc-17* 3′, which includes intron 1, and the resulting construct was pMH425. For *rab-3*, the primers were *rab-3* 5′ and *rab-3* 3′, and the resulting construct was pMH415. For *glr-1,* the primers were *glr-1* 5′ and *glr-1* 3′, and the resulting construct was pMH427. These constructs were injected and gave the expected expression except for the *unc-17* promoter*,* which had very little expression and none outside the nerve ring. To improve expression in cholinergic neurons, a different version of the *unc-17* promoter (3,656 bases in front of the ATG in exon 2) was used to generate pMH441. Neither of the *unc-17* promoter constructs, pMH441, or pMH425, include the motor neuron enhancer since this construct resulted in leaky expression in the GABA motor neurons as assayed by electrophysiology. Thus, expression in the acetylcholine motor neurons was achieved using the *acr-2* promoter. For *acr-2*, the primers were *acr-2* 5′ and *acr-2* 3′, and the resulting construct was pMH417.

### Strains.

Wild type was Bristol N2. All strains were obtained from the C. elegans Genetics Center (http://www.cbs.umn.edu/CGC) unless otherwise indicated and maintained at 22 °C on standard NGM media seeded with HB101. Strains used were: BC168, *unc-13(s69);* CB1091, *unc-13(e1091);* EG1285, *lin-15(n765)* and *oxIs12[Punc-47:GFP; lin-15(+)]*; EG1983, *unc-13(s69), unc-64(js115),* and *oxIs34[openSYX, Pmyo-2:GFP];* EG1985, *unc-64(js115)* and *oxIs34[openSYX; Pmyo-2:GFP]*; EG2279, *unc-49(e407);* EG2466, *unc-64(js115)* and *oxIs33[SYX; Punc-122:GFP]*; EG3278, *unc-64(js115)* and *oxEx536[Punc-17:SYX; Pglr-1:SYX; Punc-122:GFP; lin-15(+)];* EG3817, *unc-64(js115)* and *oxEx705[Punc-17:SYX; Pglr-1:SYX; Pacr-2:SYX; Pmyo-2:GFP];* EN560, *krIs1[Punc-47:SNB:CFP; UNC-49::YFP; lin-15(+)]* and *lin-15(n765);* MT8247, *lin-15(n765)* and *nIs52[Punc-25:SNB:GFP; lin-15(+)]*; and NM959, *unc-64(js115)/bli-5(e518)*.

To generate the acetylcholine(−) GABA(−) syntaxin mosaic strain EG3278, *unc-64(js115)* and *oxEx536[Punc-17:SYX; Pglr-1:SYX],* the strain NM959 *unc-64(js115)/bli-5(e518)* was injected using standard techniques [92] with an injection mix containing 5 ng/μl each of pMH425 and pMH427 (see Figure S1), as well as *unc-122*::GFP at 20 ng/μl (coelomocyte marker) and *lin-15(+)* at 80 ng/μl. These animals are very sick, and when maintained for long periods of time, these strains became less uncoordinated. Analysis of this derived strain demonstrated that docking was restored to 50% in the acetylcholine neurons (unpublished data). Reported data are from animals that were freshly thawed from the original isolate.

The GABA(−) syntaxin mosaic strain EG3817, *unc-64(js115)* and *oxEx705[Punc-17:SYX; Pglr-1:SYX; Pacr-2:SYX]* was generated in a similar way, except the injection mix contained pMH441, pMH417, and pMH427 (see Figure S1), as well as *myo-2*::GFP at 2 ng/μl and 1 kb ladder at 100 ng/μl (Gibco/Invitrogen, http://www.invitrogen.com). Multiple stable lines were obtained, and homozygous *unc-64* animals were recovered from each line and found to have similar phenotypes.

For fluorescence analysis of neuroanatomy in the syntaxin mosaic, strains carrying the appropriate fluorescent marker were crossed with EG3278 to generate the three strains EG3301, *unc-64(js115)/+, oxIs12,* and *oxEx536*; EG3349, *unc-64(js115)/+, nIs52,* and *oxEx536*; and EG3299, *unc-64(js115)/+, krIs1,* and *oxEx536*. Homozygous *unc-64* animals were recovered from these strains, allowed to self, and their progeny used for analysis.

### Reconstruction.

Reconstruction was performed on a VA synapse from a wild-type animal. We converted 16-bit TIFFs to 8-bit using Graphic Converter (Lemke Software GMBH, http://www.lemkesoft.com) and manually aligned using Midas (Boulder Laboratory for 3-D Electron Microscopy of Cells, University of Colorado, Boulder, Colorado, United States). The VA/VD relationship was used as a fiduciary mark during the alignment. Image segmentation was performed in 3dmod (Boulder Laboratory for 3-D Electron Microscopy of Cells) by manually tracing neuronal profiles and presynaptic specializations at 200% magnification. Synaptic vesicles were modeled as spheres with a diameter of 28 nm, and section thickness was set to 33 nm.

### Syntaxin mosaic GABA neuron morphology.

For overall neuronal morphology, ten young adult animals of each genotype (*unc-64(js115), oxIs12, oxEx536,* and wild-type *oxIs12*) were imaged on a confocal microscope and scored blind to genotype for the number of commissures. *oxIs12* expresses GFP in the GABA neurons under the control of the *unc-47* promoter. For synapse density, five young adult animals of each genotype (*unc-64(js115), nIs52, oxEx536,* and wild-type *nIs52*) were imaged on a confocal microscope. *nIs52* expresses synaptobrevin-GFP in the GABA neurons under the control of the *unc-25* promoter. For each animal, ImageJ was used to measure a region of the dorsal nerve cord, and puncta within the region were counted. For pre- and postsynaptic colocalization, ten young adult animals of each genotype (*unc-64(js115), krIs1, oxEx536,* and wild-type *krIs1*) were imaged on a confocal microscope. *krIs1* expresses synaptobrevin-CFP in the GABA neurons under the control of the *unc-47* promoter and expresses GABA_A_-receptor-YFP in muscles under the *unc-49* promoter. Colocalization of CFP and YFP was observed in all cases.

### Electron microscopy.

Previously we used ice-cold glutaraldehyde fixations for electron microscopy [73]. We have switched to high-pressure freezing followed by substitution of solvent-based fixatives [38]. Although membranes tend to be less darkly stained in this preparation, this fixation is superior to that observed with slow fixation methods. First, glutaraldehyde fixation itself stimulates exocytosis of synaptic vesicles and will therefore affect the docked pool of vesicles [75]. Second, shrinkage in conventional fixations dislodges docked vesicles and the dense projection at C. elegans synapses (our observations). Finally, changes in membrane trafficking in the coelomocytes can be observed using the slow fixation method (our observations). For these reasons we defined docked vesicles in our previous study as those within 30 nm of the plasma membrane since identifying vesicles docked at the surface was unreliable. No docking defect was observed in *unc-13* mutants using this definition [73]. Our current data using high-pressure freezing confirm this observation, since there is no significant docking defect defined by vesicles within 30 nm of the plasma membrane (number of vesicles within 30 nm, the wild type: acetylcholine = 4.57 ± 1.41, 108 profiles, GABA = 5.31 ± 1.67, 91 profiles; *unc-13(e1091)*: acetylcholine = 4.45 ± 1.17, 33 profiles, GABA = 4.53 ± 1.07, 28 profiles; *unc-13(s69)*: acetylcholine = 3.35 ± 1.30, 34 profiles, GABA = 4.16 ± 1.37, 32 profiles). Using high-pressure freezing we can now subdivide pools of docked vesicles and reliably determine if vesicles are touching the membrane; using this definition we see differences in docking in *unc-13* mutants compared to the wild type.

Worms were prepared for transmission electron microscopy essentially as described [38,93]. Briefly, ten animals were placed onto a freeze chamber (100-μm well of type A specimen carrier) containing space-filling bacteria, covered with a type B specimen carrier flat side down, and frozen instantaneously in the BAL-TEC HPM 010 (BAL-TEC, http://www.bal-tec.com). Frozen animals were fixed in a Leica EM AFS system (http://www.leica.com) with 0.5% glutaraldehyde and 0.1% tannic acid in anhydrous acetone for 4 d at −90 °C, followed by 2% osmium tetroxide in anhydrous acetone for 38.9 h with gradual temperature increases (constant temperature at −90 °C for 7 h, 5 °C/h to −25 °C over 13 h, constant temperature at −25°C for 16 h, and 10 °C/h to 4 °C over 2.9 h). Fixed animals were embedded in araldite resin (30% araldite/acetone for 4 h, 70% araldite/acetone for 5 h, 90% araldite/acetone overnight, and pure araldite for 8 h). Mutant and control blocks were blinded. Ribbons of ultrathin (33 nm) serial sections were collected using an ultracut E microtome. Images were obtained on a Hitachi H-7100 electron microscope (http://www.hitachi.com) using a Gatan (http://www.gatan.com) slow=scan digital camera. A total of 250 ultrathin contiguous sections were cut and the ventral nerve cord reconstructed from two animals representing each genotype. Image analysis was performed using ImageJ software (http://rsb.info.nih.gov/ij).

All morphometry was conducted blind to genotype and included a matched wild-type worm that was fixed in parallel. The number of synaptic vesicles (∼30 nm in diameter) in each synapse was counted, and their diameters and distances from the dense projection and plasma membrane were measured. Analysis included the acetylcholine neurons VA and VB and the GABA neuron VD.

To compare freeze-substitution fixations with our previous methods using ice-cold glutaraldehyde [73], we analyzed samples fixed previously (by W.Davis) and samples fixed recently (by S. Watanabe) and analyzed under current scoring conditions (by S. Watanabe). We observed fewer vesicles in the ice-cold glutaraldehyde fixations (average number of synaptic vesicles per profile with a dense projection, acetylcholine 7.8 SV, *n* = 28 profiles; GABA 27.7 SV, *n* = 16 profiles) compared to freeze-substituted samples (Acetylcholine 22.6 SV, *n* = 35 profiles; GABA 33.8 SV, *n* = 20 profiles).

### Electrophysiology.

Electrophysiological methods were performed as previously described [67,73] with minor adjustments. Briefly, animals were immobilized in cyanoacrylic glue (B. Braun, Aesculap, http://www.aesculapusa.com), and a lateral incision was made to expose the ventral medial body wall muscles. The preparation was then treated with collagenase (type IV; Sigma, http://www.sigmaaldrich.com) for 15 s at a concentration of 0.5 mg/ml. The muscle was then voltage clamped using the whole cell configuration at a holding potential of −60 mV. See Protocol S1 for electrophysiology solutions. GABA neuron activity was isolated by specifically blocking acetylcholine currents through the application of d-tubocurare (1 mM, Sigma) from a perfusion system. Pressure ejection of GABA from pipets of 4–5 MΩ resistance was computer triggered. Evoked responses were elicited using a fire-polished electrode positioned along the ventral nerve cord. The stimulating electrode was placed at least half a muscle length away from the patched muscle to cleanly separate the stimulus artifact from the evoked response. A square wave depolarizing current of 1 ms was then delivered from an SIU5 stimulation isolation unit driven from an S48 stimulator (Grass Telefactor, http://www.grasstechnologies.com). All recordings were made at room temperature (21 °C) using an EPC-9 patch-clamp amplifier (HEKA, http://www.heka.com) run on an ITC-16 interface (Instrutech, http://www.instrutech.com). Data were acquired using Pulse software (HEKA). All data analysis and graph preparation were performed using Pulsefit (HEKA), Mini Analysis (Synaptosoft, http://www.synaptosoft.com), and Igor Pro (Wavemetrics, http://www.wavemetrics.com). Bar graph data are presented as the mean ± S.E.M.

### d-tubocurare treatment.

To be confident about low mini rates we needed to be certain that d-tubocurare provided a complete block. d-tubocurare block was tested daily on *unc-49(e407)* to insure that the solution aliquot completely blocked acetylcholine neurotransmission. d-tubocurare was added after 2 min of recording; recordings in d-tubocurare were done for 1 min for each animal. Mini analysis was performed on the traces beginning 10 s after d-tubocurare application and on traces both before d-tubocurare application and after washout. Only those animals with full recovery after d-tubocurare washout were used. From the matched *unc-49* controls, no minis were observed in *unc-49* in 4 min of total recordings from four animals. Thus, the probability of seeing a rogue acetylcholine mini from the matched controls is less than 0.0041 fusions per second. In addition we have recorded from 103 nonmatched *unc-49* animals covering greater than an hour in d-tubocurare without seeing a single fusion event. The 0.06 fusions per second observed in the syntaxin mosaic (six minis observed) are therefore likely to be fusions from the GABA neurons. However, we cannot claim that these six minis are syntaxin-independent, since we cannot exclude the possibility that there is a low level of syntaxin expression in the GABA neurons from our transgenic array.

## Supporting Information

Figure S1 Mosaic Syntaxin Expression Constructs (A) Syntaxin is encoded by the *unc-64* locus. The plasmid pTX21 (gift of M. Nonet) rescues *unc-64*. At the 3′ end of the gene are three splice variants that encode alternative transmembrane domains (gray exons), which produce transcripts UNC-64C, UNC-64A, and UNC-64B.
(B) An *unc-64* cDNA-genomic hybrid was constructed that removes the 5′ introns but keeps the alternatively spliced 3′ exons.
(C) The minigene was placed under the control of various promoters to drive expression of UNC-64 in subsets of cells in the *unc-64* null mutant background. Each finished construct contains promoter sequence up to the ATG, an SphI site not present in wild-type syntaxin that encodes Ala-Cys and the syntaxin minigene. The *oxEx536* transgene array does not express syntaxin in the GABA motor neurons or the acetylcholine motor neurons. The *oxEx705* transgene array does not express syntaxin in the GABA motor neurons but does express it in the acetylcholine motor neurons. See Protocol S1 for primer sequences.
(105 KB PDF)

Figure S2 Expression of Syntaxin in Mosaic Animals (A) Organization of motor neuron commissures on the right side of C. elegans [94]; there are four acetylcholine commissures (green) and 16 GABA commissures (gray).
(B) Shown is antisyntaxin antibody staining of EG3278 with the *oxEx536* transgene, which lacks syntaxin in GABA and acetylcholine motor neurons. The right side of the animal is shown. There are no commissures that are syntaxin positive, consistent with lack of syntaxin in all motor neurons. Syntaxin is expressed only in the nerve ring and the head neurons that project into the ventral nerve cord. See Protocol S1 for staining procedure.
(C) Shown is antisyntaxin antibody staining of EG3817 with the *oxEx705* transgene, which lacks syntaxin in GABA neurons. The right side of the animal is shown. The position and number of commissures that are syntaxin positive is consistent with syntaxin expression in acetylcholine, but not GABA, motor neurons. Syntaxin is also expressed in the nerve ring and the sublateral cords. The green pharynx is due to GFP expression under the *myo-2* promoter (see Materials and Methods).
(753 KB PDF)

Figure S3 Synaptic Vesicles Are Distributed Normally in Syntaxin Mutant Neurons The distribution of synaptic vesicles was determined for fully reconstructed GABA synapses. Lateral axon profiles flanking the center of each dense projection (DP) were analyzed until the cloud of synaptic vesicles disappeared. These data include vesicles in the cytoplasm and docked at the plasma membrane. Because docked vesicles represent a minor portion of the total number of vesicles, this distribution largely reflects the reserve pool of vesicles.
(A) In the wild type, vesicles are most concentrated in sections containing a dense projection and form a cloud of vesicles about 700 nm in diameter.
(B) In syntaxin(−) neurons (EG3817), synaptic vesicles are distributed in a similar pattern. Distributions were centered on the center of the dense projection, and the average number of synaptic vesicles in each section determined. The average number of synaptic vesicles per axon profile is shown. Section thickness was 33 nm, so the distance from the center of the dense projection is sorted into 33-nm bins. Wild type, *n* = 10 synapses, two animals; mosaic, *n* = 5 synapses, one animal.
(225 KB PDF)

Protocol S1 Supplemental Methods (43 KB DOC)

Table S1 Comparative Statistics for Vesicle Docking (380 KB DOC)

## Abbreviations

GABA: gamma-aminobutyric acid
GFP: green fluorescent protein
SNARE: soluble N-ethylmaleimide–sensitive fusion attachment protein receptor
