# Supplementary material for: Open Syntaxin Docks Synaptic Vesicles
Source: PLoS Biol. 2007 Jul 17;5(8):e198. doi: 10.1371/journal.pbio.0050198 (PMC1914072; doi:10.1371/journal.pbio.0050198)

Supplemental Figure 1, Hammarlund et al.

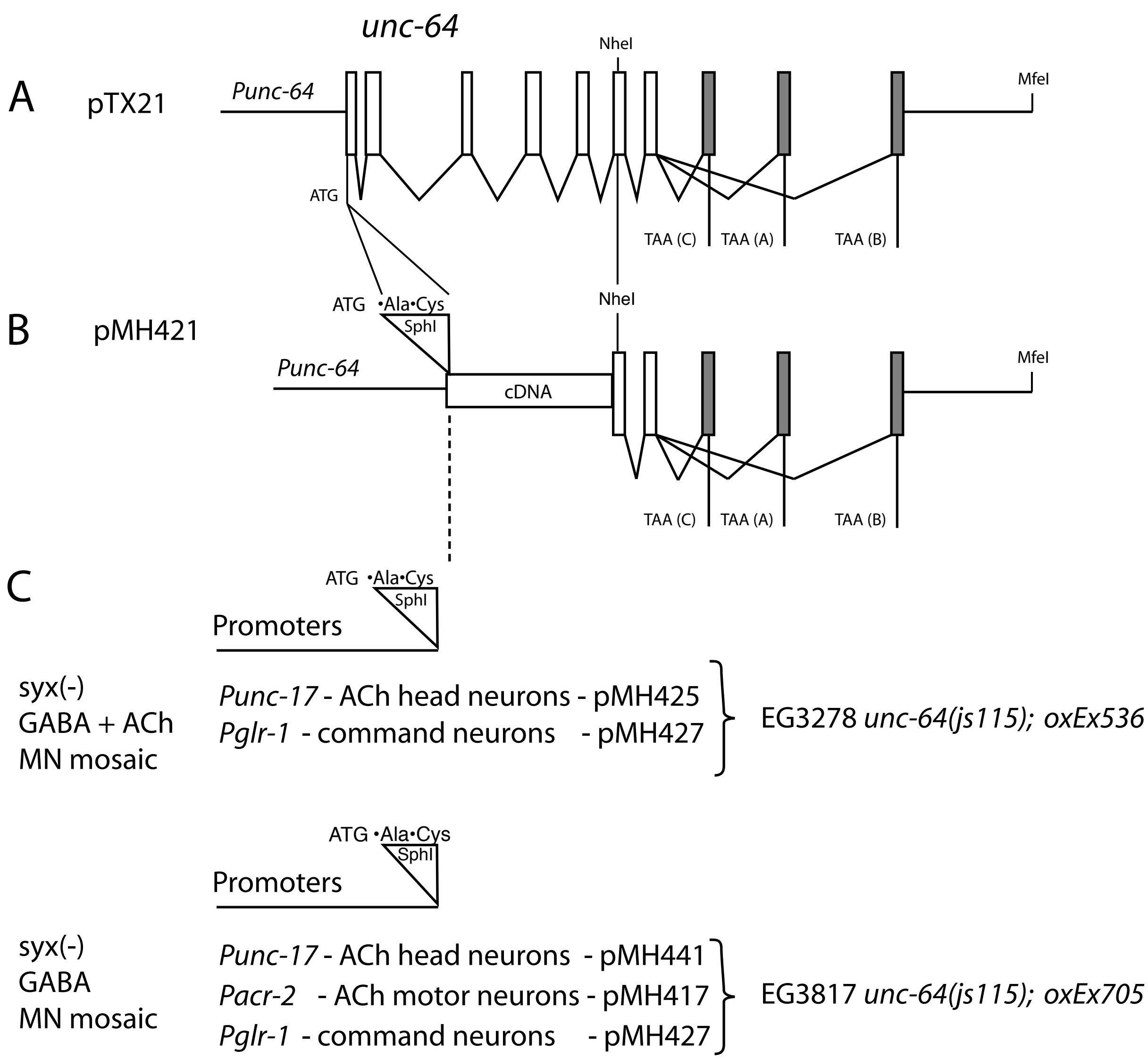

Supplement: Supplementary file 1 [file pbio.0050198.sg001.pdf]
