# Supplementary material for: Open Syntaxin Docks Synaptic Vesicles
Source: PLoS Biol. 2007 Jul 17;5(8):e198. doi: 10.1371/journal.pbio.0050198 (PMC1914072; doi:10.1371/journal.pbio.0050198)
Supplement: Supplementary file 4 [file pbio.0050198.sd001.doc]

**Supplemental methods**

**Primer sequences**

*unc-17* 5’ aagcttgcatgcctgcagg

*unc-17* 3’ ccaaaagcatgccatgcacgttgaagcccatctctc

*rab-3* 5’ gtttactagtgatcttcagatgggagcag

*rab-3* 3’ gaaagcatgccatctgaaaatagggctactgtag

*glr-1* 5’ ctcactaaagggaacaaaagctgg

*glr-1* 3’ ggaaaagcatgccattgtgaatgtgtcagattgggtgc

*acr-2* 5’ actagtgtcgacattcttggaatttcg

*acr-2* 3’ gcatgccatttttgtgcactttaagaa

**Electrophysiology solutions**

The extracellular solution contained: 150 mM NaCl, 5 mM KCl, 5 mM CaCl_2_, 1 mM MgCl_2_, 10 mM glucose, 15 mM Hepes, pH 7.35, and sucrose to 340 mOsm. The pipet solution contained: 120 mM KCl, 20 mM KOH, 4 mM MgCl_2_, 5 mM N-tris (hydroxdymethyl) methyl-2-aminoethane-sulphonic acid, 0.25 mM CaCl_2_, 4 mM NaATP, 36 mM sucrose, 5 mM EGTA, pH 7.2, sucrose to 335 mOsm.

**Syntaxin antibody staining**

Worms were prepared for staining essentially as described [1]. Briefly, animals of the appropriate genotype were freeze-cracked and fixed for 30 minutes in 4% paraformaldehyde (Electron Microscopy Sciences, #15712). Fixed worms were stained with affinity-purified anti-syntaxin antibody at 1:250 (gift of Mike Nonet), followed by anti-rabbit fluorescent-labeled secondary antibody (Molecular Probes, #A11008). Confocal images were acquired on a Zeiss 510 Meta. At least 20 well-stained animals of each genotype were examined, and the staining pattern in each case was consistent with the sample images.

**References**

1. Charlie NK, Schade MA, Thomure AM, Miller KG (2006) Presynaptic UNC-31 (CAPS) is required to activate the G alpha(s) pathway of th*e Caenorhabditis elega*ns synaptic signaling network. Genetics 172: 943-961.
