# Supplementary material for: Open Syntaxin Docks Synaptic Vesicles
Source: PLoS Biol. 2007 Jul 17;5(8):e198. doi: 10.1371/journal.pbio.0050198 (PMC1914072; doi:10.1371/journal.pbio.0050198)
Supplement: Supplementary file 5 [file pbio.0050198.st001.doc]

**Supplemental Table 1, Hammarlund *et al.***

**Comparative statistics for vesicle docking**

N = number of synapses.

P = Unpaired two-tailed t test.

Each contiguous set of serial profiles containing a dense projection was considered as a single data point. The number of docked vesicles within each such set was divided by the number of profiles in the set, resulting in a number of docked vesicles per profile for that set. A similar approach was used to calculate the number of docked vesicles per profile in each subpool (<232 nm, >330 nm, and 232-330 nm), except only vesicles at the appropriate distances were included. The mean and SEM of all data points within each genotype was determined and used to calculate P values in unpaired two-tailed t tests. Values were calculated separately for cholinergic acetylcholine and GABA synapses, as well as for all synapses considered together. For each genotype, control wild type animals were fixed in parallel and profileed, imaged and quantitated blind to genotype. Because we considered N to be the number of synapses (rather than, for instance, the number of profiles) the P values are conservative. Thus comparasions on the edge of significance (eg. *unc-13* >330 nm) may in fact represent real differences.

|  | syntaxin GABA- | | wild type | |  |
| --- | --- | --- | --- | --- | --- |
| Acetylcholine synapses | N=11 | | N=10 | |  |
|  | Mean | SEM | Mean | SEM | P value |
| profiles/synapse | 5.09 | 0.34 | 5.30 | 0.40 | 0.69 |
| docked vesicles/synapse | 15.64 | 1.20 | 15.80 | 1.42 | 0.93 |
| docked vesicles/profile | 3.09 | 0.11 | 2.99 | 0.15 | 0.59 |
| docked vesicles 0-231/profile | 2.67 | 0.11 | 2.46 | 0.18 | 0.31 |
| docked vesicles 331+/profile | 0.28 | 0.06 | 0.41 | 0.09 | 0.25 |
| docked vesicles 232-330/profile | 0.13 | 0.05 | 0.11 | 0.04 | 0.82 |
| total vesicles/profile | 26.99 | 0.77 | 27.60 | 0.78 | 0.57 |
| GABA synapses | N=10 | | N=8 | |  |
|  | Mean | SEM | Mean | SEM | P value |
| profiles/synapse | 5.10 | 0.57 | 4.75 | 0.45 | 0.65 |
| docked vesicles/synapse | 0.60 | 0.34 | 16.13 | 1.49 | 5.17E-09 |
| docked vesicles/profile | 0.09 | 0.05 | 3.42 | 0.15 | 8.30E-14 |
| docked vesicles 0-231/profile | 0.09 | 0.05 | 2.65 | 0.14 | 2.15E-12 |
| docked vesicles 331+/profile | 0.00 | 0.00 | 0.77 | 0.11 | 5.95E-07 |
| docked vesicles 232-330/profile | 0.00 | 0.00 | 0.00 | 0.00 |  |
| total vesicles/profile | 56.79 | 1.14 | 38.95 | 1.12 | 4.31E-18 |
|  | syntaxin ACh- GABA- | | wild type | |  |
| Acetylcholine synapses | N=5 | | N=5 | |  |
|  | Mean | SEM | Mean | SEM | P value |
| profiles/synapse | 4.80 | 0.86 | 4.00 | 0.32 | 0.41 |
| docked vesicles/synapse | 0.60 | 0.25 | 10.4 | 1.44 | 1.48E-04 |
| docked vesicles/profile | 0.12 | 0.05 | 2.56 | 0.22 | 4.32E-06 |
| docked vesicles 0-231/profile | 0.12 | 0.05 | 2.01 | 0.07 | 2.23E-08 |
| docked vesicles 331+/profile | 0 | 0 | 0.41 | 0.21 | 0.08 |
| docked vesicles 232-330/profile | 0 | 0 | 0.14 | 0.06 | 0.04 |
| total vesicles/profile | 19.8 | 0.94 | 21.3 | 1.64 | 0.30 |
| GABA synapses | N=4 | | N=4 | |  |
|  | Mean | SEM | Mean | SEM | P value |
| profiles/synapse | 5.50 | 0.50 | 4.00 | 0.00 | 0.02 |
| docked vesicles/synapse | 1.50 | 0.29 | 12.50 | 1.32 | 1.87E-04 |
| docked vesicles/profile | 0.27 | 0.04 | 3.13 | 0.33 | 1.39E-04 |
| docked vesicles 0-231/profile | 0.17 | 0.07 | 2.25 | 0.20 | 6.96E-05 |
| docked vesicles 331+/profile | 0.06 | 0.06 | 0.75 | 0.10 | 1.21E-03 |
| docked vesicles 232-330/profile | 0.04 | 0.04 | 0.13 | 0.07 | 0.36 |
| total vesicles/profile | 36.8 | 1.95 | 33.9 | 1.31 | 0.23 |
|  | *unc-13(s69)* | | wild type | |  |
| All synapses | N=15 | | N=22 | |  |
|  | Mean | SEM | Mean | SEM | P value |
| profiles/synapse | 4.47 | 0.46 | 4.27 | 0.44 | 0.77 |
| docked vesicles/synapse | 5.40 | 0.94 | 11.36 | 1.68 | 0.01 |
| docked vesicles/profile | 1.27 | 0.20 | 2.60 | 0.24 | 3.72E-04 |
| docked vesicles 0-231/profile | 0.31 | 0.06 | 1.91 | 0.16 | 2.18E-09 |
| docked vesicles 331+/profile | 0.85 | 0.15 | 0.41 | 0.10 | 0.01 |
| docked vesicles 232-330/profile | 0.11 | 0.04 | 0.28 | 0.06 | 0.05 |
| total vesicles/profile | 28.08 | 1.18 | 27.62 | 1.12 | 0.78 |
| Acetylcholine synapses | N=8 | | N=13 | |  |
|  | Mean | SEM | Mean | SEM | P value |
| profiles/synapse | 4.25 | 0.49 | 4.00 | 0.61 | 0.78 |
| docked vesicles/synapse | 5.00 | 1.48 | 9.46 | 2.50 | 0.21 |
| docked vesicles/profile | 1.31 | 0.37 | 2.14 | 0.25 | 0.07 |
| docked vesicles 0-231/profile | 0.35 | 0.10 | 1.69 | 0.18 | 3.32E-05 |
| docked vesicles 331+/profile | 0.82 | 0.25 | 0.19 | 0.08 | 0.01 |
| docked vesicles 232-330/profile | 0.14 | 0.07 | 0.26 | 0.09 | 0.33 |
| total vesicles/profile | 23.59 | 1.37 | 21.77 | 1.11 | 0.31 |
| GABA synapses | N=7 | | N=9 | |  |
|  | Mean | SEM | Mean | SEM | P value |
| profiles/synapse | 4.71 | 0.84 | 4.67 | 0.65 | 0.96 |
| docked vesicles/synapse | 5.86 | 1.20 | 14.11 | 1.74 | 2.55E-03 |
| docked vesicles/profile | 1.24 | 0.16 | 3.26 | 0.38 | 5.65E-04 |
| docked vesicles 0-231/profile | 0.27 | 0.07 | 2.23 | 0.26 | 1.59E-05 |
| docked vesicles 331+/profile | 0.89 | 0.14 | 0.72 | 0.16 | 0.46 |
| docked vesicles 232-330/profile | 0.08 | 0.05 | 0.31 | 0.10 | 0.08 |
| total vesicles/profile | 32.84 | 1.59 | 34.86 | 1.48 | 0.36 |
|  | *unc-13(e1091)* | | wild type | |  |
| All synapses | N=11 | | N=11 | |  |
|  | Mean | SEM | Mean | SEM | P value |
| profiles/synapse | 5.55 | 0.81 | 5.00 | 0.33 | 0.54 |
| docked vesicles/synapse | 8.91 | 1.90 | 14.36 | 1.20 | 0.03 |
| docked vesicles/profile | 1.48 | 0.14 | 2.92 | 0.21 | 1.11E-05 |
| docked vesicles 0-231/profile | 0.69 | 0.11 | 2.48 | 0.17 | 3.42E-08 |
| docked vesicles 331+/profile | 0.54 | 0.11 | 0.37 | 0.14 | 0.36 |
| docked vesicles 232-330/profile | 0.25 | 0.04 | 0.06 | 0.03 | 2.85E-03 |
| total vesicles/profile | 34.54 | 1.44 | 28.00 | 1.36 | 1.24E-03 |
| Acetylcholine synapses | N=7 | | N=7 | |  |
|  | Mean | SEM | Mean | SEM | P value |
| profiles/synapse | 4.71 | 0.78 | 5.00 | 0.38 | 0.75 |
| docked vesicles/synapse | 7.29 | 2.15 | 13.00 | 1.25 | 0.04 |
| docked vesicles/profile | 1.39 | 0.17 | 2.65 | 0.23 | 9.49E-04 |
| docked vesicles 0-231/profile | 0.65 | 0.15 | 2.57 | 0.23 | 1.27E-05 |
| docked vesicles 331+/profile | 0.50 | 0.16 | 0.08 | 0.06 | 0.03 |
| docked vesicles 232-330/profile | 0.25 | 0.06 | 0.00 | 0.00 | 1.79E-03 |
| total vesicles/profile | 31.30 | 2.07 | 22.57 | 1.07 | 4.91E-04 |
| GABA synapses | N=4 | | N=4 | |  |
|  | Mean | SEM | Mean | SEM | P value |
| profiles/synapse | 7.00 | 1.68 | 5.00 | 0.71 | 0.32 |
| docked vesicles/synapse | 11.75 | 3.59 | 16.75 | 2.17 | 0.28 |
| docked vesicles/profile | 1.63 | 0.24 | 3.39 | 0.29 | 3.39E-03 |
| docked vesicles 0-231/profile | 0.77 | 0.20 | 2.33 | 0.28 | 4.12E-03 |
| docked vesicles 331+/profile | 0.62 | 0.10 | 0.88 | 0.21 | 0.29 |
| docked vesicles 232-330/profile | 0.24 | 0.05 | 0.18 | 0.06 | 0.42 |
| total vesicles/profile | 38.36 | 1.73 | 33.82 | 2.07 | 0.10 |
|  | open syntaxin | | wild type | |  |
| All synapses | N=17 | | N=11 | |  |
|  | Mean | SEM | Mean | SEM | P value |
| profiles/synapse | 6.24 | 0.54 | 5.00 | 0.33 | 0.10 |
| docked vesicles/synapse | 20.76 | 2.48 | 14.36 | 1.20 | 0.06 |
| docked vesicles/profile | 3.27 | 0.21 | 2.92 | 0.21 | 0.27 |
| docked vesicles 0-231/profile | 2.64 | 0.16 | 2.48 | 0.17 | 0.53 |
| docked vesicles 331+/profile | 0.45 | 0.10 | 0.37 | 0.14 | 0.66 |
| docked vesicles 232-330/profile | 0.18 | 0.05 | 0.06 | 0.03 | 0.09 |
| total vesicles/profile | 30.01 | 0.95 | 28.00 | 1.36 | 0.21 |
| Acetylcholine synapses | N=10 | | N=7 | |  |
|  | Mean | SEM | Mean | SEM | P value |
| profiles/synapse | 5.70 | 0.67 | 5.00 | 0.38 | 0.43 |
| docked vesicles/synapse | 17.90 | 2.73 | 13.00 | 1.25 | 0.18 |
| docked vesicles/profile | 3.06 | 0.23 | 2.65 | 0.23 | 0.23 |
| docked vesicles 0-231/profile | 2.69 | 0.22 | 2.57 | 0.23 | 0.70 |
| docked vesicles 331+/profile | 0.21 | 0.07 | 0.08 | 0.06 | 0.20 |
| docked vesicles 232-330/profile | 0.16 | 0.07 | 0.00 | 0.00 | 0.09 |
| total vesicles/profile | 23.39 | 0.51 | 22.57 | 1.07 | 0.49 |
| GABA synapses | N=7 | | N=4 | |  |
|  | Mean | SEM | Mean | SEM | P value |
| profiles/synapse | 7.00 | 0.87 | 5.00 | 0.71 | 0.15 |
| docked vesicles/synapse | 24.86 | 4.39 | 16.75 | 2.17 | 0.22 |
| docked vesicles/profile | 3.56 | 0.39 | 3.39 | 0.29 | 0.77 |
| docked vesicles 0-231/profile | 2.56 | 0.26 | 2.33 | 0.28 | 0.58 |
| docked vesicles 331+/profile | 0.79 | 0.13 | 0.88 | 0.21 | 0.68 |
| docked vesicles 232-330/profile | 0.21 | 0.06 | 0.18 | 0.06 | 0.70 |
| total vesicles/profile | 38.43 | 1.26 | 33.82 | 2.07 | 0.06 |
|  | *unc-13* open syntaxin | | wild type | |  |
| All synapses | N=18 | | N=11 | |  |
|  | Mean | SEM | Mean | SEM | P value |
| profiles/synapse | 5.67 | 0.52 | 5.00 | 0.33 | 0.36 |
| docked vesicles/synapse | 17.22 | 1.79 | 14.36 | 1.20 | 0.26 |
| docked vesicles/profile | 3.04 | 0.13 | 2.92 | 0.21 | 0.61 |
| docked vesicles 0-231/profile | 2.18 | 0.14 | 2.48 | 0.17 | 0.19 |
| docked vesicles 331+/profile | 0.71 | 0.11 | 0.37 | 0.14 | 0.07 |
| docked vesicles 232-330/profile | 0.14 | 0.04 | 0.06 | 0.03 | 0.21 |
| total vesicles/profile | 35.20 | 1.08 | 28.00 | 1.36 | 6.00E-05 |
| Acetylcholine synapses | N=10 | | N=7 | |  |
|  | Mean | SEM | Mean | SEM | P value |
| profiles/synapse | 5.50 | 0.75 | 5.00 | 0.38 | 0.61 |
| docked vesicles/synapse | 15.60 | 2.34 | 13.00 | 1.25 | 0.40 |
| docked vesicles/profile | 2.83 | 0.12 | 2.65 | 0.23 | 0.47 |
| docked vesicles 0-231/profile | 2.15 | 0.13 | 2.57 | 0.23 | 0.11 |
| docked vesicles 331+/profile | 0.61 | 0.10 | 0.08 | 0.06 | 8.94E-04 |
| docked vesicles 232-330/profile | 0.08 | 0.03 | 0.00 | 0.00 | 0.08 |
| total vesicles/profile | 28.69 | 0.81 | 22.57 | 1.07 | 2.46E-05 |
| GABA synapses | N=8 | | N=4 | |  |
|  | Mean | SEM | Mean | SEM | P value |
| profiles/synapse | 5.88 | 0.74 | 5.00 | 0.71 | 0.47 |
| docked vesicles/synapse | 19.25 | 2.76 | 16.75 | 2.17 | 0.57 |
| docked vesicles/profile | 3.30 | 0.24 | 3.39 | 0.29 | 0.83 |
| docked vesicles 0-231/profile | 2.23 | 0.27 | 2.33 | 0.28 | 0.82 |
| docked vesicles 331+/profile | 0.85 | 0.22 | 0.88 | 0.21 | 0.92 |
| docked vesicles 232-330/profile | 0.22 | 0.08 | 0.18 | 0.06 | 0.70 |
| total vesicles/profile | 43.60 | 1.51 | 33.82 | 2.07 | 8.11E-05 |
|  | syntaxin OE | | wild type | |  |
| All synapses | N=9 | | N=9 | |  |
|  | Mean | SEM | Mean | SEM | P value |
| profiles/synapse | 3.44 | 0.41 | 4.22 | 0.49 | 0.24 |
| docked vesicles/synapse | 10.00 | 1.50 | 10.56 | 1.08 | 0.77 |
| docked vesicles/profile | 2.82 | 0.18 | 2.56 | 0.19 | 0.31 |
| docked vesicles 0-231/profile | 1.93 | 0.15 | 1.93 | 0.23 | 0.99 |
| docked vesicles 331+/profile | 0.71 | 0.15 | 0.34 | 0.11 | 0.06 |
| docked vesicles 232-330/profile | 0.19 | 0.09 | 0.29 | 0.10 | 0.44 |
| total vesicles/profile |  |  |  |  |  |
| Acetylcholine synapses | N=5 | | N=5 | |  |
|  | Mean | SEM | Mean | SEM | P value |
| profiles/synapse | 3.80 | 0.66 | 4.20 | 0.73 | 0.70 |
| docked vesicles/synapse | 10.40 | 2.23 | 11.60 | 1.17 | 0.65 |
| docked vesicles/profile | 2.67 | 0.11 | 2.89 | 0.21 | 0.38 |
| docked vesicles 0-231/profile | 2.03 | 0.11 | 2.38 | 0.22 | 0.20 |
| docked vesicles 331+/profile | 0.50 | 0.16 | 0.22 | 0.14 | 0.22 |
| docked vesicles 232-330/profile | 0.13 | 0.10 | 0.29 | 0.11 | 0.31 |
| total vesicles/profile |  |  |  |  |  |
| GABA synapses | N=4 | | N=4 | |  |
|  | Mean | SEM | Mean | SEM | P value |
| profiles/synapse | 3.00 | 0.41 | 4.25 | 0.75 | 0.19 |
| docked vesicles/synapse | 9.50 | 2.25 | 9.25 | 1.93 | 0.94 |
| docked vesicles/profile | 3.02 | 0.37 | 2.14 | 0.17 | 0.07 |
| docked vesicles 0-231/profile | 1.79 | 0.31 | 1.37 | 0.20 | 0.30 |
| docked vesicles 331+/profile | 0.98 | 0.23 | 0.49 | 0.15 | 0.12 |
| docked vesicles 232-330/profile | 0.25 | 0.16 | 0.28 | 0.19 | 0.90 |
| total vesicles/profile |  |  |  |  |  |
|  | *unc-13* syntaxin OE | | wild type | |  |
| All synapses | N=9 | | N=9 | |  |
|  | Mean | SEM | Mean | SEM | P value |
| profiles/synapse | 4.22 | 0.28 | 4.22 | 0.49 | 1.00 |
| docked vesicles/synapse | 2.44 | 0.69 | 10.56 | 1.08 | 1.01E-05 |
| docked vesicles/profile | 0.54 | 0.13 | 2.56 | 0.19 | 1.38E-07 |
| docked vesicles 0-231/profile | 0.19 | 0.05 | 1.93 | 0.23 | 1.45E-06 |
| docked vesicles 331+/profile | 0.23 | 0.08 | 0.34 | 0.11 | 0.44 |
| docked vesicles 232-330/profile | 0.12 | 0.05 | 0.29 | 0.10 | 0.13 |
| total vesicles/profile |  |  |  |  |  |
| Acetylcholine synapses | N=5 | | N=5 | |  |
|  | Mean | SEM | Mean | SEM | P value |
| profiles/synapse | 3.80 | 0.37 | 4.20 | 0.73 | 0.64 |
| docked vesicles/synapse | 1.60 | 0.68 | 11.60 | 1.17 | 7.53E-05 |
| docked vesicles/profile | 0.39 | 0.13 | 2.89 | 0.21 | 8.85E-06 |
| docked vesicles 0-231/profile | 0.22 | 0.06 | 2.38 | 0.22 | 1.31E-05 |
| docked vesicles 331+/profile | 0.13 | 0.08 | 0.22 | 0.14 | 0.60 |
| docked vesicles 232-330/profile | 0.04 | 0.04 | 0.29 | 0.11 | 0.06 |
| total vesicles/profile |  |  |  |  |  |
| GABA synapses | N=4 | | N=4 | |  |
|  | Mean | SEM | Mean | SEM | P value |
| profiles/synapse | 4.75 | 0.25 | 4.25 | 0.75 | 0.55 |
| docked vesicles/synapse | 3.50 | 1.19 | 9.25 | 1.93 | 0.04 |
| docked vesicles/profile | 0.73 | 0.23 | 2.14 | 0.17 | 2.41E-03 |
| docked vesicles 0-231/profile | 0.15 | 0.10 | 1.37 | 0.20 | 1.69E-03 |
| docked vesicles 331+/profile | 0.36 | 0.15 | 0.49 | 0.15 | 0.56 |
| docked vesicles 232-330/profile | 0.21 | 0.08 | 0.28 | 0.19 | 0.74 |
| total vesicles/profile |  |  |  |  |  |
